# Unlocking the therapeutic potential of cellular mechanobiology

**DOI:** 10.1126/sciadv.aea6817

**Published:** 2025-10-31

**Authors:** Yohalie Kalukula, Giuseppe Ciccone, Danahe Mohammed, Anthony Procès, Marie Versaevel, Amandine Deridoux, Lucie Ergot, Zoé Barbier, Maxime Mansy, Roxane Aucouturier, Rémi Tranzer, Mathieu Surin, Sylvain Gabriele, Marine Luciano

**Affiliations:** ^1^ SYMBIOSE Lab, Research Institute for Biosciences, Research Institute for Materials Science and Engineering, CIRMAP, University of Mons–UMONS, B-7000 Mons, Belgium.; ^2^ Institute for Bioengineering of Catalonia (IBEC), The Barcelona Institute for Science and Technology (BIST), Barcelona, Spain.; ^3^ Department of Molecular Biology, ULB Neuroscience Institute, Université Libre de Bruxelles (ULB), Gosselies, Belgium.; ^4^ University Grenoble Alpes, INSERM U1216, Grenoble Alpes, Grenoble Institut Neuroscience, 38000 Grenoble, France.; ^5^ Laboratory for Chemistry of Novel Materials, CIRMAP, University of Mons–UMONS, Mons, Belgium.

## Abstract

Mechanobiology is a rapidly advancing field at the intersection of biology, physics, and engineering that reveals how mechanical forces shape cellular behavior, tissue architecture, and disease progression. By elucidating how cells sense and transduce mechanical cues, mechanobiology has fundamentally advanced our understanding of processes ranging from migration and differentiation to immune responses and tissue remodeling. These advances have driven the development of innovative biophysical tools and engineered biomaterials that enable precise modulation of the cellular microenvironment. Translating mechanobiological principles into clinical practice is giving rise to mechanomedicine, a previously unrecognized paradigm that integrates mechanical forces as key modulators of health and disease. This review highlights how mechanobiology informs therapeutic strategies across diverse domains, including cancer immunotherapy, cardiovascular and neurodegenerative disorders, and regenerative medicine. By bridging fundamental discoveries with translational applications, this review positions mechanobiology as a cornerstone of next-generation medical innovation, translating mechanistic insights into impactful clinical applications.

## Teaser

Mechanical forces regulate cellular and tissue dynamics, driving disease progression and guiding next-generation therapies.

## INTRODUCTION

Recent breakthroughs in understanding how cells and tissues sense and respond to mechanical forces have propelled the emergence of mechanobiology—a multidisciplinary field at the intersection of biology, physics, and engineering (*1*). Mechanobiology explores how mechanical cues, such as stiffness, viscoelasticity, spatial confinement, curvature, and hydrodynamic forces, regulate cellular behavior and tissue architecture. These discoveries have been made possible by the development of advanced biophysical tools (*2*): from platforms that finely tune the mechanical properties of the cellular microenvironment to those enabling real-time visualization of cellular adaptation, precise control of cell spreading, confinement, and the application of mechanical forces at scales ranging from single cells to entire tissues.

Beyond mapping the structural adaptations of cells to mechanical stimuli, recent work has unraveled the signaling pathways that transduce mechanical inputs into biochemical responses—establishing a direct link between force transmission, cytoskeletal dynamics, nuclear architecture, and gene regulation (*3*). Mechanobiology has shown that transitions between distinct mechanical states are not merely passive but can actively modulate homeostasis, drive differentiation, or trigger disease. Dysregulation of these mechanical signals contributes to the pathogenesis of numerous conditions, including cancer, fibrosis, and cardiovascular and neurodegenerative diseases (*4*). This realization has catalyzed the concept of mechanomedicine—a translational framework where mechanical forces are considered both a diagnostic marker and a therapeutic target (*5*, *6*).

This review aims to present a comprehensive overview of cellular mechanobiology, focusing on the fundamental principles governing how cells sense and respond to mechanical cues. We explore the implications of these processes in disease pathogenesis and highlight recent efforts in translating mechanobiological insights into therapeutic strategies, including regenerative medicine and clinical trials.

## MECHANICAL CUES SHAPING CELL AND TISSUE BEHAVIOR

Cells and tissues are constantly exposed to a variety of mechanical cues that shape their behavior and function. These cues can arise from physicochemical modifications of the extracellular matrix (ECM) or from external mechanical forces applied to cells and tissues. These mechanical signals are sensed and integrated by cells to regulate essential processes such as migration, proliferation, differentiation, and tissue morphogenesis. Cells and tissues can also modulate their own mechanical and functional properties by activating outside-in and inside-out mechanotransduction pathways (*7*). The first part of this review focuses on the current fundamental knowledge of a selected set of mechanical cues—stiffness, viscoelasticity, curvature, spatial constraints, and hydrodynamic forces—while acknowledging that other important mechanical factors also contribute to cellular behavior (*8*).

### ECM stiffness

**Fig. 1.**
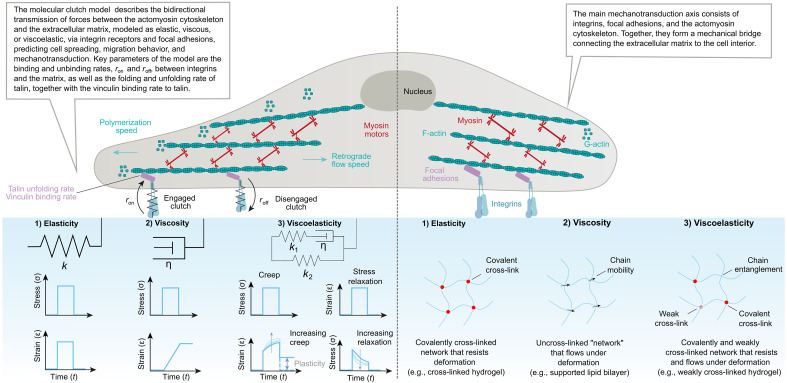
Key principles of matrix mechanics, regulatory mechanisms, and force transmission. Myosin-driven actin flow is resisted by integrins anchored to the ECM, enabling force transmission and mechanical sensing. The molecular clutch model describes how dynamic integrin engagement allows cells to detect both stiffness and viscoelasticity, with distinct responses to elastic versus time-dependent materials. Adapted from (*38*).

Among the various physical properties of the ECM, stiffness has historically emerged as a central parameter in mechanobiology. It was one of the first mechanical cues identified to directly influence cell behavior, and it remains one of the most widely studied factors in the field, although many other important cues have since been identified. The importance of matrix stiffness lies in the fact that the elastic modulus of living tissues spans several orders of magnitude. For example, brain tissues and healthy liver are very soft and exhibit elastic moduli in the range of a few hundred pascals, while muscle tissues are stiffer with an elastic modulus around 12 kPa (*9*). Tendons and cartilage, in contrast, display much higher stiffness, typically in the megapascal range (*10*). Certain tissues, such as the liver, can undergo substantial stiffening during disease progression: As fibrosis and cirrhosis develop, the elastic modulus can increase from less than 1 to ~20 kPa or even higher (*11*, *12*). In the mechanobiology literature, the terms “stiffness,” “rigidity,” and “Young’s modulus” are often used interchangeably to describe the mechanical resistance of cells and matrices, despite their subtle distinctions in physics and engineering (Fig. 1).

**Fig. 2.**
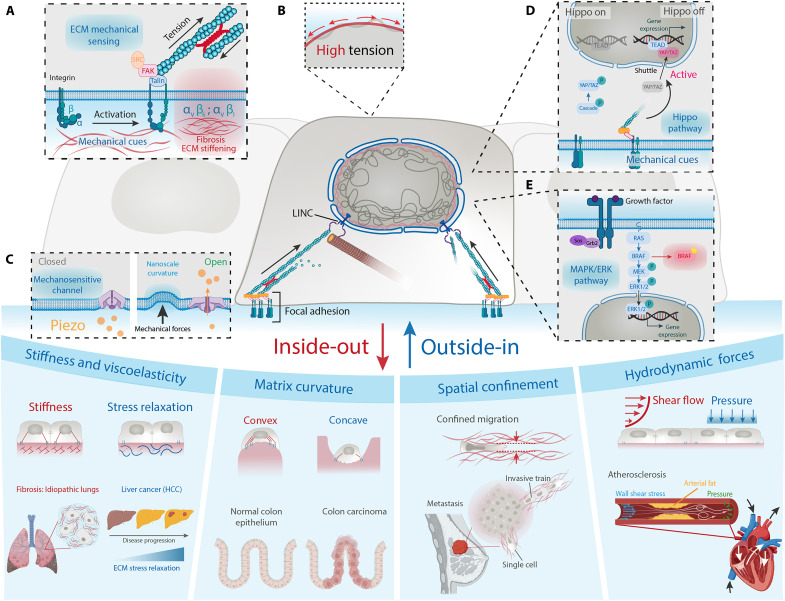
Key mechanosensing mechanisms in health and disease. (**A**) Mechanical cues activate integrins, leading to the recruitment of Talin, focal adhesion kinase (FAK), and Src and promoting actin assembly under myosin-generated tension. Specific integrins such as α_v_β_6_ and α_v_β_1_ drive profibrotic signaling and ECM stiffening. Adapted from (*66*, *143*). (**B**) Cell membrane in low (gray) and high (red) tension states, with red arrows indicating tension direction. Adapted from (*69*). (**C**) Nanoscale membrane curvature, induced by mechanical forces, promotes the opening of Piezo1 ion channels. (**D**) Hippo signaling phosphorylates Yes-associated protein (YAP)/transcriptional coactivator with PDZ-binding motif (TAZ), retaining them in the cytoplasm, while integrin-mediated signals favor nuclear localization and gene activation. Adapted from (*75*). (**E**) Receptor tyrosine kinase (RTK) activation triggers the mitogen-activated protein kinase (MAPK) pathway [Grb2–SOS–RAS–RAF–MAPK kinase (MEK)–ERK], leading to ERK1/2 nuclear entry and gene expression. In metastatic melanoma, MAPK deregulation is often deregulated by mutant BRAF. Adapted from (*83*, *199*). Bottom: Examples of extracellular mechanical cues include influencing cell behavior: increased ECM stiffness, as seen in lung fibrosis; stress relaxation, a feature of viscoelastic ECMs, elevated in liver cancer [hepatocellular carcinoma (HCC)]; matrix curvature (concave/convex topographies) affecting tissue mechanics in ductal and colorectal carcinoma; spatial confinement during single or collective cell migration in early metastasis; and shear stress and hydrostatic pressure, often disrupted in arterial narrowing during atherosclerosis. Adapted from (*19*, *53*, *144*, *151*, *160*).

A foundational insight from mechanobiology is that the stiffness of standard tissue culture substrates, such as plastic or glass, lies in the gigapascal range, which is orders of magnitude stiffer than the native cellular environment. As a result, most cells are cultured under nonphysiological mechanical conditions. The recognition of this mismatch has helped explain the high failure rate of in vitro studies, particularly pharmacological tests, which often lead to misleading conclusions and poor translation to clinical settings (*13*). A second key realization regarding the importance of mechanical conditions is that modulating the mechanical properties of the cellular environment is a critical factor for the establishment and maintenance of proper cellular and tissue functions (Fig. 2).

Over the past few decades, extensive research has elucidated how cells and tissues sense and respond to variations in ECM stiffness (*14*). These efforts have been supported by the development of tunable polymeric matrices, advanced biochemical functionalization strategies, force-based assays, and nanomechanical tools capable of probing forces down to the molecular scale (*15*). In a landmark study, mesenchymal stem cells (MSCs) were cultured on polyacrylamide substrates with varying stiffness to investigate how matrix mechanics influence stem cell differentiation. The results demonstrated that MSCs differentiated into neurocytes on soft matrices (0.1 to 1 kPa), skeletal muscle cells on intermediate matrices (8 to 17 kPa), and osteoblasts on stiff matrices (>34 kPa), underscoring the pivotal role of matrix stiffness in directing cell lineage specification. Together, these advances have revealed the remarkable mechanosensitivity of cells and tissues, showing that key cellular functions, such as migration, proliferation, and differentiation, are optimized within a specific range of ECM stiffness (Fig. 2).

Very recent studies have unveiled previously unidentified molecular mechanisms by which cells transduce mechanical signals into biological outcomes. For example, a specific region within the talin protein, known as the R1R2 interface, acts as a critical force–sensitive switch that enables cells to sense and adapt to the stiffness of their surrounding environment (*16*). Destabilizing this interface lowers the threshold for focal adhesion formation, allowing cells to spread and generate traction forces even on soft matrices. This finding identifies a direct mechanotransduction pathway linking matrix mechanics to cytoskeletal organization and tissue integrity. Talin, a key protein in cell adhesion, controls this connection through its interaction with actin-related protein 2/3 complex subunit 5-like (ARPC5L), as part of the machinery that builds actin fibers. This reveals a previously unknown layer of regulation in how cells respond to their environment.

Together, these studies illustrate how fundamental mechanobiology continues to reveal intricate pathways by which cells sense, integrate, and respond to the mechanical properties of their environment. While much attention has focused on stiffness, it is crucial to recognize that the ECM is not a purely elastic material: It exhibits viscoelastic and plastic properties, such as stress relaxation and creep, which also play critical roles in tissue homeostasis and disease progression (*17*–*19*).

### ECM viscoelasticity

Although the first synthetic polymer matrices, such as polyacrylamide hydrogels, successfully reproduced the variability of ECM stiffness (*14*, *20*), they still failed to capture its native mechanical complexity. The initial substrates developed for mechanobiology studies behaved as linear elastic solids under cellular deformations, whereas natural ECMs exhibit time- and deformation-dependent mechanical behaviors, including nonlinear elasticity, stress relaxation, creep, and plasticity (Fig. 1).

Rheological analyses reveal that in most soft tissues—such as liver, breast, muscle, skin, and adipose tissue—the loss modulus (viscous component) is typically about 10 to 20% of the storage modulus (elastic component) when measured at 1 Hz (*21*). This indicates that, although these tissues are primarily elastic, they also display a substantial capacity for viscous dissipation. Stress relaxation experiments show that these soft tissues gradually lose their mechanical resistance to deformation, typically over timescales ranging from tens to hundreds of seconds (*22*, *23*). This relaxation is essential for adapting to dynamic forces without accumulating damage. In idiopathic pulmonary fibrosis and liver cancer, altered ECM stress relaxation perturbs tissue mechanics and activates mechanotransduction pathways that drive disease progression (Fig. 2). Notably, even stiffer tissues—such as bone, tendon, ligaments, and cartilage—exhibit viscoelastic behavior, with a loss modulus of ~10% of the storage modulus, underscoring that viscoelasticity is not exclusive to soft tissues.

These viscoelastic properties arise from the weakly cross-linked, hydrated protein networks that make up the ECM (*21*, *24*, *25*). For example, collagen networks resist deformation via cross-links, which determines their elastic modulus, but the unbinding and reorganization of weak noncovalent interactions dissipate energy over time, producing stress relaxation and creep, which determines their viscoelastic properties (*26*). Under sustained forces, the ECM can undergo plastic deformations, resulting in irreversible changes to its structure. Consequently, natural ECMs such as collagen and reconstituted basement membrane (e.g., Matrigel) are viscoplastic materials (*27*).

In biological tissues such as collagen networks, viscoelastic relaxation enables the formation of transient mechanical gradients that guide the movement of groups of cells, even without chemical signals to direct them (*28*). This property is not limited to natural tissues. Engineered materials with similar viscoelastic behaviors have been shown to enhance the development of functional blood vessels and promote healing after heart injury (*29*). On a smaller scale, the way cells attach to and pull on their surroundings using transmembrane proteins (integrins and cadherins) is also influenced by viscoelasticity (Fig. 2). This can change how cells sense forces and make decisions about their future, such as whether to become bone or fat cells (*30*, *31*). Moreover, the combination of viscoelasticity and physical confinement, such as squeezing cells into tight spaces, can further affect how cells spread and migrate, as seen in models of breast epithelial cells (*32*).

Understanding how ECM properties (e.g., stiffness and viscoelasticity) modulate cytoskeleton-dependent processes such as cell migration has prompted considerable theoretical interest in the role of adhesion complexes. These structures, which act collectively as molecular clutches, ensure mechanical coupling between the actin cytoskeleton and the ECM through transmembrane receptors. The motor-clutch model describes how cells transmit forces to their surrounding matrix through dynamic interactions between actomyosin contractility and adhesion complexes (Fig. 1) (*33*). Clutch components, composed of mechanosensitive proteins, undergo conformational changes when stretched, entering distinct functional states that initiate specific biochemical signals. These signaling outputs are shaped not only by the magnitude of force but also by the temporal characteristics—such as duration, frequency, and mechanical history—of each force transmission event. In adhesion-dependent migration, cells often exhibit a biphasic response to substrate stiffness (*34*), with maximal traction and motility at intermediate stiffness. Some cells, however, overcome this constraint by reinforcing adhesions in a force-dependent manner (*35*), allowing sustained migration even on stiff matrices. Beyond stiffness, viscoelasticity of the ECM plays a crucial regulatory role: Molecular slippage within clutch complexes and viscous stress dissipation influence force transmission (*36*). Notably, fast stress-relaxing soft substrates can enhance clutch engagement and promote faster cell migration (*37*), highlighting the importance of both elastic and viscous matrix properties in guiding cell behavior (Fig. 2). Collectively, these findings underscore that viscoelasticity is not a passive property but an active regulator of cellular mechanotransduction, migration, and differentiation, shaping cell fate decisions across physiological and pathological contexts (*38*).

### Matrix curvature

Beyond the variation in mechanical properties, tissues and organs exhibit intrinsic curvature at both their outer and inner interfaces, which profoundly influences cellular organization and behavior (*39*). These curvatures, which can be either concave or convex, vary in magnitude across tissues, typically ranging from 10^−4^ to 1 μm^−1^ (*39*). An increasing body of evidence highlights local curvature as a key parameter of the cellular microenvironment, regulating cell morphology, polarity, and function (Fig. 2).

Curved surfaces generate spatially varying stress fields: Convex regions promote tension accumulation, while concave zones tend to dissipate mechanical forces. Cells interpret these cues through a dynamic balance of forces arising from cytoskeletal contractility, osmotic pressure, and membrane tension. The actomyosin cytoskeleton reorganizes in response, forming distinct architectures: Stress fibers align along concave boundaries (*40*), while lamellipodia and branched actin networks preferentially localize to convex areas (*41*). Microtubules and actin filaments act in concert to generate compressive and tensile forces that shape both individual cells and multicellular tissues. These mechanical adaptations are propagated across tissue scales via adhesive structures such as focal adhesions and cadherin-based junctions, enabling collective cellular responses to curvature. In vitro studies have shown that migrating cells are guided by the curvature of their substrate—a phenomenon known as curvotaxis (*42*). Cells exhibit preferential migration along specific curvatures, driven by the minimization of membrane-substrate adhesion energy and the energetics of membrane protein complex binding (*43*). At the nuclear level, curvature induces shape deformations that influence gene expression: Nuclei are compressed vertically at convex interfaces, which can alter nuclear pore organization and the localization of mechanosensitive transcriptional regulators, while concave regions relieve mechanical constraints (*39*, *44*).

While these observations support a central role for curvature in cellular mechanosensing, the precise molecular mechanisms by which cells detect and transduce curvature cues remain an active area of investigation (*39*). Nonetheless, curvature-driven mechanical gradients have been shown to regulate cell fate decisions, promoting osteogenesis on convex zones and adipogenesis in concave regions (*45*, *46*). Together, these findings illustrate how cells integrate geometrical and mechanical cues through curvature sensing to regulate cytoskeletal organization, nuclear architecture, and gene expression, which are processes increasingly implicated in pathological contexts such as fibrosis, cancer progression, and tissue remodeling.

### Confinement and spatial constraints

Matrix curvature can locally modulate confinement and spatial constraints by increasing cellular density within specific regions, but spatial constraints are more broadly imposed by the architecture of the tissue and the dimensions of the extracellular space. High cellular density in compact tissues and limited matrix dimensions create spatial restrictions that shape cell migration, invasion, and cellular energy metabolism.

In single and collective cell migration, substrate adhesion and integrin-mediated frictional forces emerge as pivotal regulators (*47*). At the single-cell level, spatial confinement reduces the available adhesion area and alters protrusive forces, thereby directly modulating migration speed (Fig. 2) (*48*). Spatial confinement induces lamellipodial thickening, which, in turn, reduces actin treadmilling and lowers protrusive force generation, ultimately slowing cell migration speed (*48*). Cell trains—linear chains of single cells—are particularly efficient at navigating complex microenvironments, aided by microtubule alignment along the migration axis and the formation of cryptic lamellipodia that facilitate forward movement (*49*). In contrast, larger cell clusters that exhibit extensive cell-cell adhesions accumulate internal stresses that propagate transversely to the migration direction, slowing down collective movement, whereas interaxial contacts appear to play a minimal role (*49*).

Spatial confinement has also emerged as a potent mechanical cue that regulates cell behavior through nuclear deformation. When cells migrate through spaces smaller than their nuclear diameter, the nucleus stretches and unfolds its envelope, triggering a cascade of mechanotransductive events. This deformation activates stretch-sensitive pathways that increase actomyosin contractility, enabling cells to adjust their motility in confined environments (*50*). In vivo studies in zebrafish further show that nuclear envelope unfolding under confinement elevates intracellular calcium levels, which remodel actomyosin networks and promote migration plasticity (*51*). Together, these findings position the nucleus as a central mechanosensor that interprets spatial constraints to control cytoskeletal dynamics and guide cellular adaptation in crowded three-dimensional (3D) tissues.

Beyond migration, spatial confinement has been shown to directly influence stem cell fate. Recent work using physiologically relevant elastomer-based microchannel systems revealed that confined migration alone, without any additional biochemical cues, can promote osteogenic differentiation in human MSCs (*52*). Cells that navigated through narrow 3- to 5-μm channels exhibited persistent nuclear deformation, increased levels of acetylated histones—an indicator of gene activation—and up-regulated genes associated with bone formation, all of which are early markers of osteogenic differentiation. These findings highlight that confined migration can act as a potent mechanical cue capable of initiating differentiation pathways through deformation-induced nuclear mechanotransduction. Together, this body of fundamental mechanobiology research provides critical insights into why highly polarized and persistent tumor clusters composed of up to eight cells are frequently observed in patients with epithelial-originating cancers (*53*–*55*) and highlights the pivotal role of spatial confinement in regulating both physiological and pathological processes.

### Hydrodynamic forces and shear stresses

In addition to the modulation of the physicochemical properties of the ECM and the spatial constraints imposed by the surrounding tissue architecture, cells and tissues are also continuously subjected to external forces, such as fluid shear stress and pressure, which profoundly affect cellular behavior and tissue homeostasis (Fig. 2). This is, for example, the case of endothelial cells (ECs), which line the interior surface of blood vessels and continually experience shear stress from laminar blood flow and pressure gradients.

In response to laminar shear stress, ECs undergo morphological changes, elongating and aligning their long axis in the direction of flow (*56*, *57*). This reorientation is accompanied by cytoskeletal remodeling, including the alignment of actin filaments into stress fibers oriented with the flow, providing structural support and facilitating signal transduction mainly to orient and adopt elongated shape (*58*). Microtubules also align along the flow direction, contributing to intracellular transport and maintaining cell polarity (*59*). Cell-cell junctions, particularly those involving vascular endothelial (VE)–cadherin, are dynamically remodeled under shear stress to maintain vascular integrity. These junctions redistribute and strengthen in response to flow, ensuring barrier function while allowing for necessary permeability.

A recent controversy has emerged regarding the role of cell-cell junctions in endothelial mechanotransduction. While some studies claimed that ECs can align to shear flow without cell-cell contacts, multiple independent laboratories have demonstrated that cell-cell junctions are essential for shear stress sensing and alignment over physiologically relevant timescales (*60*). Cells in blood vessels use specialized proteins such as platelet endothelial cell adhesion molecule 1, vascular endothelial growth factor receptor 2 (VEGFR2), and VE-cadherin to sense mechanical forces such as blood flow. These proteins help convert these physical forces into chemical signals inside the cell, which, in turn, control gene expression and cellular behavior to maintain vascular homeostasis (*61*).

The cellular response to shear stress exemplifies the intricate integration of mechanosensing pathways, a critical area of study, as dysregulated shear stress is increasingly recognized as a key driver of human diseases, including atherosclerosis, aneurysm formation, thrombosis, and cancer metastasis (*62*, *63*). Understanding how cells sense and respond to hydrodynamic forces is essential to grasp the profound impact of these mechanical cues on tissue physiology and pathology. Together, the molecular and biophysical mechanisms of mechanosensing provide a fundamental framework that allows cells to adapt to their dynamic mechanical environment. These principles will be explored in greater detail in the next section.

## CELLULAR AND MOLECULAR MECHANISMS OF MECHANOSENSING

Cells have evolved sophisticated molecular machinery to sense and respond to the mechanical forces of their environment. As shown previously, ECs, for example, rely on specialized mechanosensors to perceive shear stress and pressure exerted by blood flow (*64*). This section focuses on the key components of cellular mechanosensing and introduces representative pathways that link mechanical cues to gene expression, cell fate, and disease.

### Integrins and focal adhesions are physical anchors and signaling hubs

At the core of this mechanosensing machinery are integrins, heterodimeric transmembrane receptors that physically and functionally bridge the ECM and the cytoskeleton (*65*). Each cell type expresses a tailored set of integrin heterodimers—selected from among the 24 available heterodimers—that are specifically tuned to recognize and bind distinct ECM proteins, thereby integrating mechanical information with the biochemical composition of their microenvironment. By forming focal adhesions, which are dynamic multiprotein assemblies, integrins enable cells to sense, transmit, and exert forces across the plasma membrane (Fig. 2A). Mechanical cues, such as the cues discussed in the previous sections, are then converted into biochemical signals through downstream pathways involving focal adhesion kinase (FAK) and Src family kinases, a group of enzymes that regulate how cells grow, move, and respond to their surroundings (*66*). This system enables two-way communication between external mechanical forces and internal biochemical signals.

### Cytoskeletal connectivity and linker of nucleoskeleton and cytoskeleton complexes

Beyond the cell membrane, integrins connect to the cytoskeleton, composed of actin filaments, microtubules, and intermediate filaments. The cytoskeleton itself connects to the nuclear envelope via LINC (linker of nucleoskeleton and cytoskeleton) complexes, creating a continuous mechanical axis that spans from the extracellular environment to the nucleus, where genetic material is stored (*44*). Through this architecture, contractile forces generated by actin filaments—powered by myosin motor proteins—can be transmitted across the cell and ultimately relayed to the ECM.

### Membrane tension is a global modulator of mechanosensitivity

Membrane tension plays a crucial role in regulating integrin engagement and clustering (Fig. 2B) (*67*, *68*). Acting as a global integrator of mechanical signals, membrane tension sets the threshold for integrin activation, enabling cells to dynamically adapt their mechanotransduction responses to fluctuations in their physical environment (*69*). Together, integrins, cytoskeletal elements, LINC complexes, and membrane tension establish an integrated, hierarchical system for mechanosensing and mechanotransduction (*70*).

While these membrane-associated structures and pathways provide an essential framework for mechanosensing, recent discoveries have expanded our understanding of how cells decode mechanical signals through noncanonical mechanisms. Beyond classical signaling pathways, cells also rely on a diverse array of molecular sensors, including ion channels, G protein–coupled receptors, and mechanically responsive transcription factors, that broaden the landscape of mechanotransduction (*4*).

In the following sections, we highlight three representative mechanosensitive pathways, Piezo1, Yes-associated protein (YAP)/transcriptional coactivator with PDZ-binding motif (TAZ), and mitogen-activated protein kinase (MAPK)/extracellular signal–regulated kinase (ERK), to illustrate how cells interpret and respond to mechanical cues through distinct molecular strategies. Together, these examples highlight the diversity and sophistication of mechanosensitive signaling and their relevance to essential processes such as migration, differentiation, and fate decisions, which are fundamental drivers of both physiological regulation and disease.

### Piezo1 is a nanoscale sensor of force and geometry

Piezo1 is an essential mechanosensor that translates physical cues from the cellular microenvironment—such as matrix stiffness, viscoelasticity, and mechanical forces—into biochemical signals that regulate cell behavior (Fig. 2C). This mechanosensitive ion channel allows the entry of calcium (Ca^2+^) in cytoplasm to modulate signaling pathways and chemical signals. Recent studies have largely expanded our understanding of Piezo1’s role in mechanobiology. Notably, Piezo1’s spatial distribution is regulated by membrane curvature: It is excluded from highly curved membrane protrusions such as filopodia but enriched in nanoscale membrane invaginations, suggesting that Piezo1 functions as a nanoscale sensor of the cell’s geometric landscape (*71*). This curvature-dependent localization underscores its role as an integrator of both mechanical forces and membrane topology. Piezo1 activation—triggered by chemical agonists such as Yoda1 or by mechanical stretching—can lead to its redistribution across the membrane, reinforcing its function as a dynamic mechanosensory. Beyond its role in sensing, Piezo1 also modulates cytoskeletal organization and cell migration, notably by regulating filopodia dynamics and interacting with cytoskeletal components in a force-dependent manner (*72*). Recent work has further demonstrated that Piezo1 mediates cellular responses to matrix viscoelasticity in MSCs, promoting cell spreading, focal adhesion formation, cytoskeletal remodeling, and nuclear tension in a stiffness-dependent manner within viscoelastic microenvironments (*73*). Piezo1-dependent mechanotransduction also influences critical processes such as mitochondrial dynamics and energy metabolism, linking mechanical forces to bioenergetic regulation. Piezo1 activity modulates the nuclear localization of YAP, thereby controlling gene expression programs essential for stem cell fate decisions (*74*).

### YAP/TAZ are transcriptional coactivators of mechanical inputs

YAP and TAZ are transcriptional coactivators classically associated with the Hippo pathway, where they regulate gene expression programs that regulate cell proliferation, survival, and differentiation (Fig. 2D). However, YAP/TAZ can also be activated by mechanical cues, including substrate stiffness, confinement and stretch, independently of their canonical Hippo signaling context (*75*). Acting as central mechanotransducers, they integrate physical signals from the cellular microenvironment to control gene expression patterns that govern critical processes including migration, differentiation, and cell fate decisions (*75*). Notably, YAP plays a crucial role in maintaining tissue homeostasis. In epithelial monolayers, cells exposed to higher mechanical strain undergo nuclear deformation, enhancing YAP nuclear localization and triggering localized compensatory proliferation (*76*). These spatial differences in nuclear mechanics and mechanical input contribute to region-specific transcriptional responses that help preserve epithelial integrity.

In the context of cell migration, YAP promotes directed movement during embryonic development. In gastrulating fish embryos, YAP sustains dorsal migration and midline convergence by coordinating actomyosin tension and focal adhesion dynamics, ensuring proper axis formation (*77*). In cancer models, YAP facilitates invasive migration by orchestrating a Rho–guanosine triphosphatase switch: It promotes Rac1 activation while suppressing RhoA/ROCK (Rho-associated coiled-coil containing protein kinase) signaling, thereby enhancing cell motility across diverse systems including glioblastoma and epithelial cells (*78*). In cell differentiation, YAP activity is tightly linked to mechanical context. For instance, in skeletal muscle differentiation, myoblast elongation leads to YAP cytoplasmic retention and nuclear export, facilitating differentiation into myotubes (*79*). In stem cells, YAP dynamically modulates cell fate: Optogenetic manipulation of YAP levels in embryonic stem cells revealed that pulsatile YAP activity enhances pluripotency markers such as octamer-binding transcription factor 4 (Oct4) and proliferation, while sustained YAP repression promotes differentiation (*80*). Moreover, substrate curvature can influence YAP distribution through nuclear deformation, modulating chromatin organization and proliferation in epithelial monolayers (*81*). Lastly, YAP integrates mechanical cues from the ECM, with its nuclear translocation modulated by viscoelastic dissipation. Even on stiff matrices, high energy dissipation blunts YAP activation, revealing a nuanced interplay between rigidity and viscosity in cell mechanosensing (*82*).

### The MAPK/ERK pathway integrates mechanical cues into classical signaling

Finally, the MAPK/ERK pathway, traditionally associated with growth factor signaling, has emerged as a key mechanosensitive module. In the classical model, ERK sits at the core of the MAPK cascade, a signaling pathway that governs critical cellular processes such as apoptosis, migration, and mitosis (Fig. 2E) (*83*, *84*). The pathway is typically initiated by receptor tyrosine kinase (RTK) activation—often triggered by growth factors such as epidermal growth factor (EGF)—which sets off a phosphorylation cascade culminating in ERK activation. Once phosphorylated, ERK translocates into the nucleus, where it modulates gene expression programs that drive diverse cellular outcomes (Fig. 2E). While pulsatile ERK activity is known to vary depending on the specific cellular function being regulated (*84*, *85*), recent studies have revealed the notable mechanosensitivity of the pathway (*86*, *87*). For instance, during collective cell migration, mechanical stretching of leader cells activates ERK through EGF receptor signaling, generating unidirectional ERK waves that propagate to follower cells and coordinate collective motion (*86*). Similarly, in tissue repair, mechanical stretching of cells in opposite directions triggers ERK activation, promoting regenerative responses in mouse skin models (*88*).

**Fig. 3.**
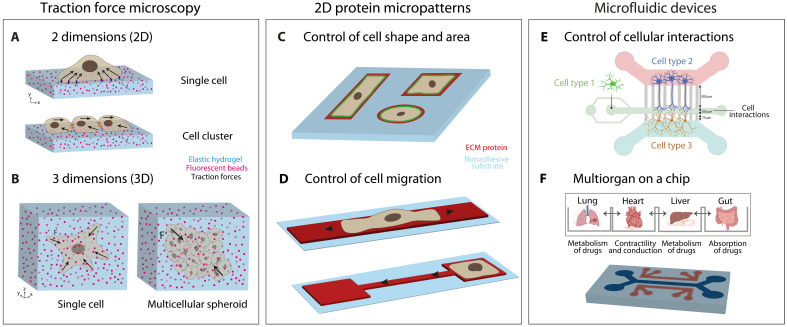
Biophysical and material tools to decode mechanobiology. (**A**) 2D traction force microscopy (TFM) allows to quantify cell traction forces at the interface between an elastic hydrogel (in blue) and cells seeded on top of it by tracking the displacement of fluorescent beads embedded within the hydrogel. The displacement is reconstructed into forces via computational methods. (**B**) 3D TFM is similar to 2D TFM; however, it is applied to cells embedded within hydrogels rather than seeded on top of them. (**C** and **D**) 2D protein micropatterns are produced by different techniques, such as microcontact printing or ultraviolet (UV)–based methods, with the aim to precisely generate geometrically defined ECM protein islands (in red) on different materials, such as glass, elastomers, and hydrogels (in blue). Protein micropatterns can be used to control (C) cell shape and area or (D) cell migration (*113*). Microfluidic devices are usually produced by bonding microfabricated devices made of elastomeric material to glass coverslips. (**E**) These devices can be used to impose and study dynamic cellular deformations or generating multicellular compartments to study heterogeneous cellular interactions. Adapted from (*124*). (**F**) Multiorgan on a chip devices used in personalized medicine applications.

Together, integrins, the cytoskeleton, membrane tension, Piezo1, YAP/TAZ, and MAPK/ERK constitute central hubs within the cellular mechanotransduction network. These pathways convert mechanical inputs into molecular signals that orchestrate cell behavior, fate decisions, and disease outcomes. Understanding how these pathways operate in different contexts not only deepens our knowledge of cell biology but also opens promising avenues for translational applications in mechanomedicine. To fully explore these pathways and their relevance in health and disease, it is essential to employ biophysical tools capable of precisely manipulating and quantifying mechanical cues (Fig. 3).

## DECODING MECHANOBIOLOGY: BIOPHYSICAL TOOLS AND TRANSLATIONAL APPLICATIONS

Substantial efforts have focused on developing tools to modulate the physicochemical properties of the cellular environment, measure rheological properties of cells and tissues, replicate mechanical constraints, and assess contractile forces at both single-cell and multicellular levels. These technologies have become indispensable for dissecting the physical forces that shape cellular behavior and for bridging the gap between mechanobiological insight and translational applications. Techniques include micropipette aspiration, microneedles, micropatterning, microfluidics, magnetic tweezers, traction force microscopy (TFM), atomic force microscopy, particle-tracking microrheology, and others (*89*). Together, these approaches offer a comprehensive toolkit for probing mechanical behavior across scales. More recently, quantitative phase imaging (*90*) and Brillouin microscopy (*91*) have emerged as powerful, label-free, and noncontact methods to study cellular and tissue viscoelastic properties. These different tools have advanced our understanding of mechanobiology and, in some cases, have been adapted for biomedical applications. This section highlights selected key tools, major insights, and their potential for translational research.

### TFM for quantifying cell-generated forces

TFM has become a pivotal technique for quantifying cellular forces and understanding how cells sense and respond to mechanical cues. Since its introduction by Dembo and Wang in 1999 (*92*), TFM enables precise measurement of cellular tractions (force per unit area) on compliant substrates embedded with fluorescent markers (Fig. 3A). By imaging substrates in relaxed and tensed states and using particle image velocimetry—now available through open-source implementations (*93*)—TFM has been widely adopted. It has been applied to diverse processes, such as cardiomyocyte beating (*94*), epithelial cell migration guided by physicochemical footprints (*95*), and the regulation of molecular clutch dynamics (*96*). Originally, 2D TFM algorithms have been adapted to compute full 3D stress fields (Fig. 3B) (*97*, *98*). For instance, 3D TFM revealed how altered ECs guide neighboring cells by exerting pulling forces and reshaping the surrounding matrix (*99*). Beyond fundamental insights, 2D and 3D TFM have been applied to pathological contexts. TFM has shown that increased stiffness in breast tumors amplifies cellular traction forces, promoting nuclear deformation, invasion, and metastasis (*100*). Moreover, 3D TFM quantified the collective forces of tumor spheroids in nonlinear collagen matrices (*101*), demonstrating how collagen stiffening mediates mechanical feedback and drives cancer invasion, highlighting the critical role of 3D mechanical forces in tumor progression.

### Protein micropatterns for shaping cellular geometry and function

TFM outputs are inherently sensitive to cellular parameters such as adhesion area and cell geometry. This has led to increasing interest in combining TFM with physicochemical approaches to control these variables, particularly on soft culture substrates where deformation fields can be visualized. A pivotal advance came from Whitesides’ (*102*) adaptation of microcontact printing for biological applications, enabling the creation of micrometer-scale protein islands with predefined shapes and sizes (Fig. 3, C and D). This technique allows precise control over cell spreading area, specific ECM interactions, and overall geometry (*103*). Since its introduction, microcontact printing has evolved into more robust methods, such as ultraviolet (UV)–based protein patterning (*104*, *105*), which supports functionalization of soft hydrogels for TFM (*106*), the creation of protein gradients (*107*), and complex biochemical microenvironments (*108*). Because of these advances, protein micropatterning has become widely accessible, with commercial systems available for easy adoption in biology laboratories (*105*). Protein micropatterns have transformed mechanobiology, revealing fundamental processes such as, for instance, the relationship between cell shape (*109*) and function (*110*) (Fig. 3C), centrosome positioning (*111*), migration in confined spaces (*48*, *49*, *112*–*114*) (Fig. 3D), myoblast fusion (*79*), and contractile force repair relative to cytoskeletal geometry (*115*, *116*). Beyond fundamental research, micropatterns are now being applied in translational contexts. For example, multicolor protein micropatterns on nanoporous silica substrates have been used for multiplexed immunoassays, enabling simultaneous detection of multiple antigens (*117*). High-throughput platforms using micropatterned 96-well plates allow systematic analysis of cell behavior under various stimuli, aiding drug discovery and toxicology testing (*118*). In tissue engineering and regenerative medicine, micropatterns guide stem cell differentiation and tissue formation (*119*, *120*).

### Microfluidic platforms for controlling confinement and nuclear mechanics

However, the influence of the microenvironment extends beyond the cell periphery and mechanical forces are also transmitted to the nucleus, where they regulate genome organization and gene expression. To probe the mechanics of the nucleus itself, complementary techniques have emerged, including microfluidic devices that quantify nuclear viscoelasticity across diverse cell types, for instance, in tumor cells (*121*) and induced pluripotent stem cells (iPSCs) (*122*). These studies revealed that nuclear mechanics vary across cell types and disease states, linking nuclear stiffness to cell fate and pathology. For immune cells, microfluidic platforms have shown how cytoskeletal dynamics regulate leukocyte trafficking through narrow capillaries, with actin governing deformability and myosin II modulating surface wrinkling and shape relaxation (*123*). In the nervous system, microfluidics has enabled studies of microglial responses to mechanical stress: Compartmentalized devices have reconstructed corticocortical networks, revealing how microglia-neuron interactions and synaptic dynamics adapt to mechanical cues (Fig. 3E) (*124*). A microfluidic artery model demonstrated that ECs respond to pulsatile flow, shear stress, and cyclic strain in a stiffness-dependent manner, linking pathological mechanical cues to impaired barrier function and proatherogenic responses (*125*). Beyond fundamental studies, microfluidics is advancing translational research. It has revealed how nuclear deformability is altered in diseases such as laminopathies, with implications for cell migration and differentiation (*122*). High-throughput microfluidic micropipette assays have also identified how force duration influences spheroid deformability (*126*). Lastly, single-cell mechanical phenotyping of dissociated tissue biopsies highlights microfluidics’ potential in clinical applications such as cancer diagnostics, therapeutic monitoring, and tissue engineering (*127*).

### From bench to bedside: Emerging tools for mechanomedicine

These biophysical techniques, along with many others, are in continuous development to address increasingly specific needs in translational applications. For example, microfluidic systems have led to the emergence of organ-on-chip technologies, which aim to replicate the microarchitecture and function of human organs (*128*). These systems are now widely used in drug development and disease modeling, with ongoing efforts to integrate multiple organ systems to simulate whole-body physiology (Fig. 3F) (*129*). Brillouin microscopy, a noninvasive, label-free optical technique, has also become a powerful tool for measuring the viscoelastic properties of cells and tissues at high resolution (*130*). This technique is being explored in clinical contexts, including the assessment of corneal stiffness in ophthalmology and the mechanical characterization of tumors (*131*, *132*). Last, high-intensity focused ultrasound (HIFU) has emerged as a noninvasive therapeutic modality that uses focused ultrasound waves to ablate diseased tissue (*133*). HIFU has already been applied in clinical settings for the treatment of conditions such as prostate cancer and uterine fibroids, demonstrating its effectiveness in targeted tissue ablation (*134*). Together, these examples illustrate the successful translation of biophysical tools from fundamental mechanobiology into clinical and therapeutic applications. These advances have not only transformed our understanding of cellular and nuclear mechanobiology but also laid the groundwork for exploring how mechanical forces contribute to human disease.

## MECHANOBIOLOGY AND DISEASE PATHOGENESIS: TOWARD MECHANOMEDICINE

Building on fundamental knowledge gained from cellular mechanobiology, the identification of key signaling pathways, and the development of various tools and platforms, many studies in the field have shifted toward the investigation of human diseases, giving rise to the term “mechanomedicine.”

### ECM stiffness and viscoelasticity shape cancer progression

**Fig. 4.**
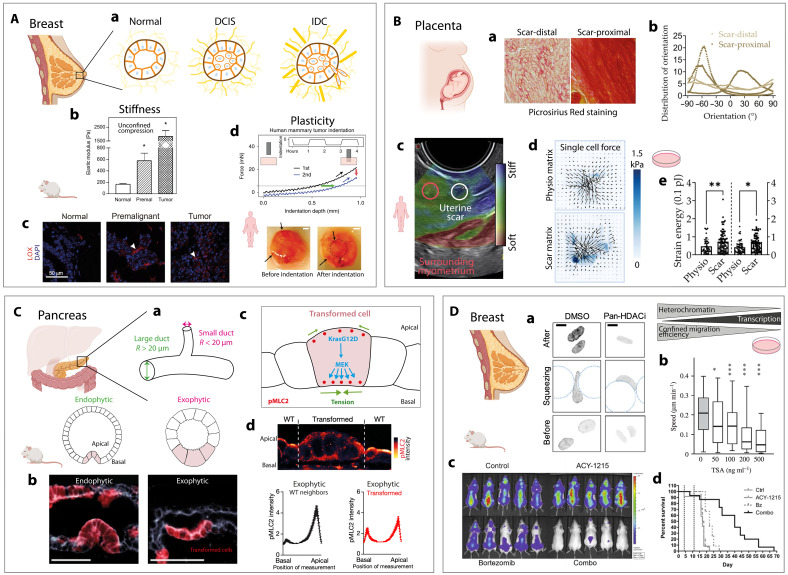
Mechanobiology in disease pathogenesis. Created in BioRender, S.G. (2025) (https://BioRender.com/31hwpic). (**A**) Breast tumor progression. (a) Mammary duct progression: normal (left), DCIS (middle), and IDC (right). Yellow fibers indicate collagen. (b) Elastic modulus in a mouse model: normal, premalignant (DCIS), and malignant (IDC) tissues. (c) Confocal images of tissues in (b) stained for LOX (red) and 4′,6-diamidino-2-phenylindole (DAPI) (nuclei; blue). (b) and (c) Adapted from (*135*). (d) Force-indentation curves highlight plastic deformation of human mammary tumors. Images of samples pre- and postindentation highlight plastically deformed regions (arrows). Scale bars, 1 mm. Adapted from (*136*). (**B**) Placenta accreta spectrum (PAS). (a) Picrosirius Red staining of PAS tissue (scar-proximal versus scar-distal). (b) Collagen fiber orientation distributions in patients with PAS. (c) Uterine scar stiffness in women prior Cesarean section by ultrasound elastography. Adapted from (*241*). (d) Traction force map of decidualized endometrial stromal fibroblasts on physiological (top) and scar (bottom) matrices. (e) Quantification of cell strain energy and energy density. (a), (b), (d), and (e) Adapted from (*140*). (**C**) Mechanical deformation in ducts and colon carcinoma. (a) Large ducts (>20 μm) show exophytic deformation, and small ducts (<20 μm) show endophytic deformation. (b) Exophytic and endophytic deformations in KFCk19 mice. Scale bars, 50 μm. (c) Summary of mechanical changes in transformed cells. (d) Basal-apical phosphorylated myosin light chain 2 (pMLC2) intensity profiles in wild-type (WT) neighbors and transformed cells for an exophytic deformation. Adapted from (*148*). (**D**) Spatial confinement influences cancer invasion. (a) H3K27me3 in HT1080 cells treated with dimethyl sulfoxide (DMSO) (control) or pan–HDAC inhibitor (HDACi) during confined migration. Scale bars, 10 μm. Adapted from (*153*). (b) Mean cell migration efficacy over 24 hours with increasing trichostatin A (TSA). Adapted from (*152*). (c) Bioluminescent images of mice treated with saline, ACY-1215, bortezomib (Bz), or ACY-1215 + bortezomib for 2 weeks. (d) Survival quantification for treatments. (c) and (d) Adapted from (*155*). *P ≤ 0.05, **P ≤ 0.01, and ***P ≤ 0.001.

The ECM is a dynamic and complex scaffold whose mechanical properties vary widely across tissues and change over time during development, aging, and disease. A hallmark of many cancers, including breast cancer (Fig. 4A), is progressive ECM stiffening, primarily driven by enhanced collagen deposition and cross-linking. This stiffening promotes tumor progression by activating integrin-mediated mechanotransduction pathways, which regulate cell adhesion, migration, and survival. For example, during the transition from ductal carcinoma in situ (DCIS) to invasive ductal carcinoma (IDC), ECM stiffening creates a proinvasive microenvironment, facilitating tumor cell invasion and dissemination (Fig. 4A, a to c) (*135*). While matrix stiffening is often accompanied by enzymatic remodeling via matrix metalloproteinases (MMPs), recent studies showed that viscoelastic and plastic ECM properties can independently drive cancer invasion, even without important biochemical degradation (*135*). Increased ECM plasticity allows tumor cells to deform and migrate through mechanically restrictive environments, promoting metastasis to distant sites (Fig. 4Ad) (*136*). A key molecular contributor to ECM stiffening is lysyl oxidase (LOX), a copper-dependent enzyme that catalyzes covalent cross-linking of collagen fibers by oxidizing lysine and hydroxylysine residues in collagen’s telopeptide domains (*137*). Elevated LOX expression is frequently observed in tumors, where its activity increases tissue stiffness and disrupts normal tissue function (Fig. 4Ac) (*138*, *139*). Conversely, inhibiting LOX activity has been shown to reduce ECM stiffness and limit fibrosis and tumor progression (*11*). Beyond stiffness alone, ECM viscoelasticity, particularly stress relaxation rate and viscoplastic behavior, have emerged as a critical regulator of cell behavior. Faster stress relaxation promotes malignant phenotypes by allowing cells to remodel their microenvironment more efficiently, supporting processes such as epithelial-to-mesenchymal transition and metastatic dissemination (*18*). These mechanical changes directly influence key signaling pathways, including YAP/TAZ activation, highlighting the tight coupling between ECM mechanics and cancer progression.

### ECM-driven, fibrosis, and scar mechanics

Fibrotic diseases are paradigmatic examples of pathological mechanotransduction. In placenta accreta spectrum (PAS) (Fig. 4B), a severe obstetric disorder marked by abnormal placental invasion, histological analyses of scar tissue from prior uterine surgeries have revealed disorganized collagen architecture (Fig. 4B, a and b) and increased stiffness—two key features of impaired ECM remodeling (Fig. 4B, c to e) (*140*). This stiffened microenvironment promotes excessive cellular contractility (Fig. 4B, d and e), driven by Rho/ROCK and YAP/TAZ signaling. This mechanical stress also activates Piezo1 channels, leading to inflammatory responses that further contribute to PAS pathogenesis. Together, fiber misalignment and increased stiffness disrupt mechanical feedback between maternal and placental tissues, promoting invasive trophoblast behavior and poor decidualization. Emerging biophysical models suggest that altered scar mechanics reduce tissue resistance to placental anchoring, exacerbating invasion (*140*). These findings highlight the central role of altered biomechanics and mechanosensitive pathways—particularly Piezo1-mediated inflammation—in PAS, emphasizing the need to incorporate tissue mechanics into clinical risk assessment and future therapeutic strategies.

Similarly, fibrosis in general is driven by sustained myofibroblast activation, excessive ECM deposition, and aberrant integrin engagement. A central mediator of this process is transforming growth factor–β (TGF-β), a master regulator of fibrogenesis that is stored in the ECM in a latent form. Mechanical tension applied via specific integrins, such as α_v_β_6_ and α_v_β_1_ (Fig. 2A), activates latent TGF-β (*141*), leading to downstream signaling that perpetuates fibroblast activity and ECM stiffening. This mechanosensitive activation mechanism links cellular contractility to fibrotic progression and has made integrins and TGF-β signaling attractive therapeutic targets (*142*). Inhibitors targeting TGF-β receptors or integrin-mediated activation (e.g., α_v_ integrin antagonists) are currently under clinical investigation in various fibrotic diseases, including idiopathic pulmonary fibrosis (*143*, *144*) and liver fibrosis (*145*). Recent studies further highlight that the reversal of fibrosis may require not only biochemical inhibition but also mechanical reprogramming. The transition of myofibroblasts back to a quiescent state is controlled by dynamic changes in fibronectin tension, integrin usage, and expression of matrix remodeling enzymes (*146*). These insights underscore the importance of therapies that disrupt maladaptive force transmission and mechanically driven TGF-β activation, offering original strategies to halt or reverse fibrotic progression.

### Tissue curvature drives cancer progression and prognosis

Emerging evidence underscores the pivotal role of tissue curvature in cancer development and progression. Interfacial curvature has been identified as a critical mechanobiological parameter influencing tumor morphogenesis and cellular behavior (*147*, *148*). In pancreatic cancer models (Fig. 4C), studies using micropatterned hydrogels have demonstrated that convex curvature enhances edge stress, promoting a myofibroblast-like phenotype in cancer-associated fibroblasts (CAFs) (*149*). Similarly, in melanoma, convex curvature at the tumor periphery has been shown to prime cancer stem cells (CSCs), the subpopulation responsible for metastasis and recurrence, with CSCs preferentially localizing to these regions (*150*). Clinically, the significance of curvature is further highlighted in colon adenocarcinoma. Analysis of biopsies from 97 patients revealed that advanced cancer stages exhibit increased interfacial curvature, correlating with poorer prognosis, higher recurrence rates, and reduced survival. These findings suggest that local curvature not only serves as a mechanobiological cue but also holds potential as a prognostic biomarker (*151*). Further emphasizing the role of curvature, in vivo and histological studies have shown that local epithelial curvature influences tumorigenesis (*148*). In the pancreatic ductal system, curvature thresholds dictate lesion expansion direction, either outward (exophytic) or inward (endophytic) (Fig. 4C, a and b). Transformed cells in both cases exhibit apical-basal redistribution of phosphorylated myosin light chain 2 (pMLC2) (Fig. 4C, c and d), indicating conserved cytoskeletal remodeling. Notably, a critical tube curvature of ~0.05 μm^−1^, corresponding to a radius of 20 μm, has been identified as the threshold for this transition across various organs and cancer types (*148*). Collectively, these insights reveal that tissue curvature is not merely a geometric feature but an active regulator of cellular behavior and disease progression, offering innovative avenues for prognostic assessment and therapeutic intervention in oncology.

### Spatial confinement influences cancer cell invasion

Several strategies have been developed to limit confined migration and thereby reduce the invasive potential of tumor cells, for instance, in breast tissues (Fig. 4D). One promising approach involves the pharmacological stabilization of microtubules through inhibition of histone deacetylase 6 (HDAC6). Trichostatin A (TSA), a pan–HDAC inhibitor, has been shown to reduce migration through 3D confining pores and impair cancer cell dissemination (Fig. 4Da) (*152*, *153*). While TSA effectively inhibits HDAC6 and reduces cancer cell migration speed (Fig. 4Db), its broad inhibition of multiple HDAC isoforms raises concerns about off-target and pleiotropic effects. These studies suggest that HDAC6 activity plays a key role in enabling tumor cells to adapt to physical confinement and sustain invasive migration under restrictive conditions. In addition, TSA affects chromatin structure and gene expression globally, complicating its use in vivo. Its poor pharmacokinetic profile and cytotoxicity at higher doses further limit its clinical applicability. These challenges have driven the development of more selective HDAC6 inhibitors—such as ricolinostat (ACY-1215), currently under clinical evaluation—with better safety and efficacy profiles (*154*). It was, for instance, demonstrated in preclinical studies using mouse xenograft models that ACY-1215 exhibited antitumor activity both as a single agent and in combination with bortezomib, a proteasome inhibitor Fig. 4Dc (*155*). At low doses, the combination of ACY-1215 and bortezomib produced a synergistic anti–multiple myeloma (MM) effect. In vivo, the anti-MM efficacy of the combination was further validated in two different severe combined immunodeficient mouse xenograft models, where median overall survival was significantly extended in animals receiving the combination therapy compared to those treated with either agent alone (Fig. 4Dd).

Metastatic cell clusters exhibit elevated integrin tension at their trailing edge, enabling them to navigate confined microenvironments such as narrow tissue spaces or microchannels (*156*). However, confinement imposes substantial energetic demands, driving metabolic reprogramming in migrating cells. This adaptation is characterized by an increased adenosine 5′-triphosphate (ATP)/adenosine 5′-diphosphate ratio and heightened glucose uptake—metabolic shifts that fuel persistent migration under physical constraints. To conserve energy, cells preferentially migrate along paths of least resistance. Cell deformability plays a critical role: Softer cells exhibit enhanced invasive capacity in confined environments. These findings underscore the intricate coupling between mechanical constraints, cellular mechanics, and metabolism, highlighting the potential of targeting altered metabolic pathways as a strategy to restrict metastatic cell migration (*157*).

### Mechanical forces fueling cancer progression, invasion, and metastasis

**Fig. 5.**
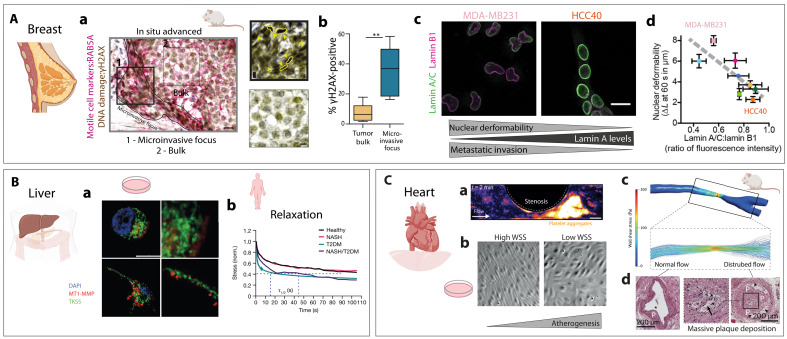
Mechanobiology in disease pathogenesis. Created in BioRender, S.G. (2025) (https://BioRender.com/31hwpic). (**A**) Biophysical profiling for breast cancer. (a) Human breast tumors stained for RAB5A (red) and γH2AX (brown). Scale bars, 80 and 25 μm (inset). Yellow arrowheads mark deformed nuclei. (b) Quantification of γH2AX-positive nuclei shows greater DNA damage in invasive foci than in the tumor bulk. **P ≤ 0.01. (a) and (b) Adapted from (*158*). (c) Lamin A/C and lamin B1 immunolabeling for MDA-MB231 and HCC40 cells. Scale bar, 20 μm. (d) Nuclear deformability inversely correlates with the lamin A/C:lamin B1 ratio. Colored dots indicate a panel of breast cancer cell lines. (c) and (d) Adapted from (*159*). (**B**) Matrix viscoelasticity in liver cancer. (a) tyrosine kinase substrate with 5 SH3 domains (TKS5) (green) and membrane type 1 MMP (MT1-MMP) (red) staining of HCC cells in fast-relaxing hydrogels mimicking diseased liver showing enhanced invasive protrusions (bottom) versus slow-relaxing hydrogels (top). Scale bar, 10 μm. (b) Stress relaxation curves across liver disease progression: healthy, nonalcoholic steatohepatitis (NASH), and type 2 diabetes mellitus (T2DM). (a) and (b) Adapted from (*19*). (**C**) Vascular stenosis and atherosclerosis. (a) Confocal microscopy of platelet aggregates on ECs, platelets labeled using 3,3′-dihexyloxacarbocyanine iodide (DiOC 6; false color). Scale bar, 100 μm. Adapted from (*166*). (b) Endothelial response to wall shear stress (WSS): High WSS induces elongation and flow alignment, whereas low WSS causes disordered organization, inflammation, and permeability, hallmarks of early atherogenesis. (c) Computational fluid dynamics of a vascular cuff, showing low laminar shear stress upstream and low oscillatory shear stress downstream. (b) and (c) Adapted from (*160*). (d) Carotid artery cross sections with a cuff: Laminar shear stress proximally prevents atherosclerotic plaque, while oscillatory shear stress downstream promotes plaque (P) with lipid core (black arrows). Vessel lumen was indicated by an asterisk. Adapted from (*63*, *242*).

Cancer progression is intimately linked to mechanical cues within the tumor microenvironment. In breast cancer, nuclear deformability has emerged as a promising biomarker of metastatic potential (Fig. 5A). Histological analyses of breast tumors reveal that microinvasive regions, which are rich in motile cell markers such as Ras-related protein Rab-5A (RAB5A) and display elevated DNA damage [phosphorylated form of histone H2A variant X (γH2AX)], contain a high density of deformed nuclei, typically associated with cell crowding and migration (Fig. 5A, a and b) (*158*). These findings suggest a strong correlation between nuclear deformation and invasive behavior. Supporting this, a recent study analyzing breast cancer biopsies reported a direct link between low lamin A expression, increased nuclear deformability, and poor patient prognosis (Fig. 5A, c and d) (*159*).

Beyond nuclear mechanics, the viscoelastic properties of the ECM also play a critical role in shaping cancer cell behavior. Recent studies highlight the impact of ECM stress relaxation—independent of stiffness—on liver disease progression, notably under conditions such as nonalcoholic steatohepatitis (NASH), type 2 diabetes mellitus (T2DM), and hepatocellular carcinoma (HCC) (*19*). Under these conditions, the accumulation of advanced glycation end-products alters ECM stress relaxation, promoting cancer cell proliferation and survival by activating mechanotransduction pathways, such as YAP/TAZ signaling via Tensin 1 in vivo (Fig. 5B). Experimental models using mechanically engineered high viscoelastic hydrogels, designed to mimic the malignant in vivo environment, have revealed further insights into how ECM viscoelasticity drives cancer invasion. Cells cultured within these matrices form invadopodia, which are specialized, linear protrusions that facilitate ECM degradation and invasion. Invadopodia are characterized by canonical markers such as membrane type 1 MMP (MT1-MMP), tyrosine kinase substrate with 5 SH3 domains (TKS5), active integrin β_1_, and pMLC (Fig. 5Ba). In addition, stress relaxation curves showed that the tissue relaxes quickly in the pathogenic liver, meaning that internal stress decreases efficiently over time as observed in soft, viscoelastic behavior, while the curves show faster stress relaxation as the liver progresses toward NASH and T2DM, indicating decreased mechanical resistance and altered matrix viscoelasticity (Fig. 5Bb). This mechanical time-dependent softening and increased ability to dissipate stress are thought to create a microenvironment that favors tumor initiation and progression, even before fibrotic scarring becomes clinically apparent. These findings highlight the critical role of matrix mechanics in regulating tumor progression, providing a compelling rationale for targeting the mechanical properties of the tumor microenvironment as part of therapeutic strategies.

### Mechanical stress and vascular pathology

Variations in shear stress across the vascular network strongly influence EC behavior and can trigger disease onset and progression (Fig. 5C). In regions of low or disturbed shear stress, such as arterial branches and bifurcations, aberrant activation of developmental signaling pathways leads to inflammation, increased permeability, and atherosclerotic plaque formation, key features of early atherogenesis (Fig. 5C, a and b) (*63*). More broadly, mechanical homeostasis, when dysregulated, contributes to pathologies including hypertension, atherosclerosis, arrhythmias, and preeclampsia (*160*, *161*). In atherosclerosis, for example, endothelial damage triggers inflammatory cascades and the accumulation of lipids and other substances in the vessel wall, leading to plaque formation, arterial narrowing, and impaired blood flow. Recent advances in mechanobiology have identified mechanical cues as critical regulators of EC function, opening promising therapeutic avenues. Microstretcher platforms mimicking hypertensive stress reveal how ECs adapt to mechanical overload (*162*–*164*), offering insights into endocytosis-based drug delivery, while VEGFR2 emerges as a key mechanochemical sensor and therapeutic target in flow-related vascular diseases (*58*, *165*). Complementary in vitro models, such as microfluidic stenotic chambers, have proven essential for replicating shear-dependent vascular processes, including thrombus formation (*166*–*168*). These systems reveal how platelets preferentially aggregate downstream of high-occlusion sites, where flow separation and recirculation zones create localized low shear regions that favor thrombus development (Fig. 5Ca) (*166*). Beyond thrombosis, these models have been instrumental in demonstrating how spatial variations in wall shear stress (WSS) influence EC behavior. Under high WSS, ECs align parallel to the flow direction, promoting vascular quiescence and barrier integrity. In contrast, regions of low or oscillatory WSS—common downstream of stenotic sites—induce random EC organization, increased permeability, and proinflammatory gene expression, contributing to early atherogenic events (Fig. 5Cb) (*160*, *161*). Clinically, integrating endothelial shear stress measurements with coronary computed tomography (CT) angiography now enables noninvasive mapping of shear stress distributions, improving the prediction of myocardial perfusion abnormalities and aiding in risk stratification (Fig. 5Cc) (*169*). These disturbed flow regions often correlate with sites of massive atherosclerotic plaque deposition, as observed in zones of turbulent flow within coronary arteries (Fig. 5Cd). Lastly, bridging mechanobiology with clinical research—such as ongoing trials targeting mechanosensitive signaling pathways such as p38 MAPK, which are implicated in lamin-related cardiomyopathies (*170*, *171*)—holds great promise for advancing cardiovascular therapies and improving patient outcomes.

### Mechanical forces and neurodegenerative disorders

**Fig. 6.**
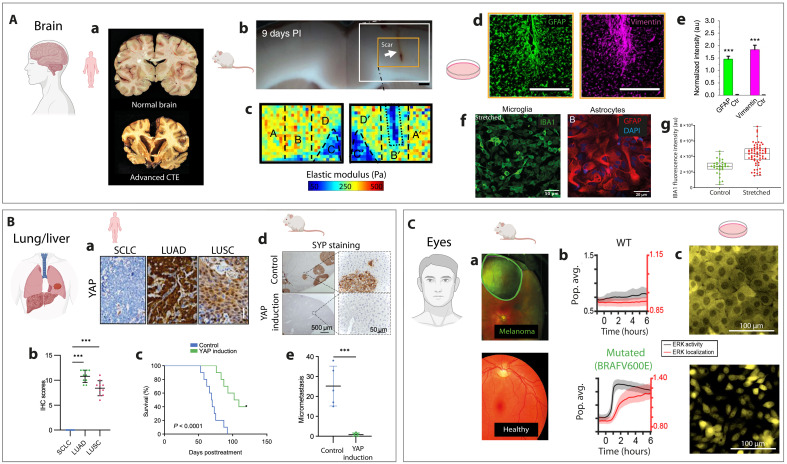
Mechanobiology of brain injury, cancer progression, and metastasis. Created in BioRender, S.G. (2025) (https://BioRender.com/31hwpic). (**A**) TBI and CTE. (a) Repetitive mild TBI leads to CTE, characterized by brain weight reduction, cortical atrophy, and enlarged ventricles. Adapted from (*173*). (b) Image of glial scar 9 days postinjury (PI). Scale bar, 2 mm. (c) Tissue stiffness map showing reduced tissue stiffness ipsilateral to the lesion. (d) Immunofluorescence showing increased GFAP (astrocytes; green) and vimentin (purple) around the lesion. Scale bars, 500 μm. (e) Quantification of GFAP/vimentin expression, highlighting reactive gliosis. au, arbitrary units. (b) to (e) Adapted from (*182*). (f) Immunofluorescence of stretched cells showing increased IBA1 (microglia; green) and GFAP (astrocytes; red). (g) Quantification of IBA1 increases after 20% stretch. (f) and (g) Adapted from (*124*). (**B**) *YAP* silencing in SCLC and its antimetastatic effects upon reexpression. (a) YAP protein detection by immunostaining in lung adenocarcinoma (LUAD), lung squamous carcinoma (LUSC), and small cell lung carcinoma (SCLC) tumors. (b) Quantification of immunohistochemical (IHC) score confirms high YAP expression in LUAD and LUSC but absent in SCLC. (c) Survival curve of nude mice after orthotopic injection of control or YAP inducible H209 cells. (d) Liver sections from vehicle and doxycycline-treated mice, stained against synaptophysin (SYP), demonstrating that YAP induction inhibits liver metastasis (brown areas). (e) Quantification of liver metastasis (foci per mouse) following treatment. Adapted from (*192*). (**C**) MAPK pathway deregulation in metastatic uveal melanoma. (a) Eye fundus images showing healthy eye tissue versus uveal melanoma. Adapted from (*203*). (b) BRAF V600E mutation drives MAPK pathway hyperactivation, with pulsatile dynamic and sustained ERK activity observed over 6 hours. (c) Fluorescence imaging of ERK dynamics using an ERK-mRuby2 reporter in MCF10A cells, showing increased nuclear recruitment of ERK in MAPK-mutated cells (bottom) compared to healthy controls (top). (b) and (c) Adapted from (*200*). ***P ≤ 0.001.

Traumatic brain injury (TBI) exemplifies a disease where mechanical forces directly induce brain deformation (*172*), resulting in internal stress and inflammation (Fig. 6A). Depending on the magnitude and location of the mechanical insult, TBI predisposes individuals to neurodegenerative diseases such as Alzheimer’s, Parkinson’s, and amyotrophic lateral sclerosis. Repeated concussions, often leading to chronic traumatic encephalopathy (CTE) (Fig. 6Aa), are characterized by brain atrophy, particularly in the frontal and temporal lobes, and a progressive inflammatory response driven by glial cells (*173*). This secondary injury mechanism highlights the brain’s specific vulnerability to mechanical stress due to its rheological properties and limited adaptive capacity (*174*). Unlike other tissues, the brain cannot adapt to short-term mechanical changes, making it especially vulnerable to injury (*175*). Studies emphasize the importance of the mechanical environment on neural cells (*176*, *177*) and their own rheological properties (*178*, *179*). Neurons, astrocytes, oligodendrocytes, and microglia are all sensitive to mechanical stress, which can inhibit axonal regrowth and modulate inflammatory signaling pathways. For instance, mechanical loading has been shown to influence neuronal fate (*180*, *181*), while glial scars formed by astrocytes after injury soften brain tissue (Fig. 6A, b and c) and reorganize the ECM (Fig. 6Ac) (*182*), impeding regeneration. Increased glial fibrillary acidic protein (GFAP) and vimentin expression have been observed around scar lesions (*182*), consistent with reactive gliosis (Fig. 6A, d and e). Axonal growth, sensitive to stiffness gradients (*179*), further underscores the critical role of brain tissue mechanics in development and repair. In vitro studies replicating TBI-like mechanical conditions have demonstrated that single or repetitive uniaxial (*124*, *183*) and biaxial (*184*, *185*) deformations induce inflammatory states in glial cells. Astrocytes subjected to stretch exhibit increased inflammatory signaling, influencing synaptic modulation through tumor necrosis factor–α receptor isoforms (*186*). Studies on microglial cells revealed that stretch injury increases ionized calcium-binding adapter molecule 1 (Iba1) protein levels, densifies actin cytoskeleton, and enhances migratory persistence (Fig. 6A, f and g) (*124*). These cytoskeletal and migratory adaptations are consistent with previous findings implicating the involvement of the integrin/FAK pathway in microglial reactivity to mechanical stretch (*176*, *185*). Mechanically activated microglial cells introduced into healthy cortical neuronal networks in microfluidic devices demonstrated synaptic pruning activity independently of neuronal injury. These insights provide a more comprehensive understanding on the role of microglial cells in synaptic loss following TBI (*187*).

### YAP/TAZ is a mechanosensitive transcriptional regulator in disease

YAP/TAZ act as key mechanosensitive transcriptional regulators across a wide spectrum of diseases. Recent studies highlight how nuclear deformation and chromatin tension directly regulate YAP activity, positioning nuclear mechanics as a central determinant of YAP-mediated mechanotransduction (*188*). Within the nucleus, YAP/TAZ bind to TEA domain family member 1 (TEAD) transcription factors and activate gene programs that are dysregulated across a spectrum of diseases, including cancer (*189*), cardiac dysfunction (*190*), and immune disorders (*191*).

In lung and liver cancers, YAP’s role in tumor progression and metastasis appears notably context dependent (Fig. 6B). Immunohistochemical (IHC) analysis revealed that YAP protein is highly expressed in lung adenocarcinoma (LUAD) and lung squamous cell carcinoma (LUSC) but absent in small cell lung cancer (SCLC) (Fig. 6B, a and b) (*192*). This was confirmed by quantitative analysis of patient samples, underscoring YAP’s selective role in non-SCLCs. Functionally, orthotopic mouse models demonstrated that inducing YAP expression in H209 cells—derived from the bone marrow of a patient with SCLC—significantly prolonged mouse survival in orthopedic models (Fig. 6Bc) and reduced liver metastases, as shown by histology and immunostaining after doxycycline treatment (Fig. 6B, d and e). These findings suggest that YAP activation can suppress SCLC metastatic potential, emphasizing the context-specific effects of YAP signaling on tumor progression.

Similarly, in breast cancer, YAP has been identified as a central regulator of collective invasion, operating through a positive mechanotransduction feedback loop. YAP activation enhances cytoskeletal tension and ECM remodeling via transcriptional up-regulation of actomyosin contractility and fibronectin production, which, in turn, further stimulates YAP signaling in leader cells. Disruption of YAP or its downstream effectors impairs force generation, ECM alignment, and collective invasion, highlighting YAP’s pivotal role in coordinating mechanical and transcriptional programs that drive tumor progression (*193*). Beyond cancer, YAP/TAZ signaling also regulates key processes in post–myocardial infarction remodeling, including cardiomyocyte proliferation, fibroblast activation, and angiogenesis (*194*). While YAP hyperactivation is often linked to tumor progression, such as in malignant pleural mesothelioma (*195*), recent findings also reveal YAP’s tumor-suppressive roles within the tumor microenvironment. For instance, excessive ECM deposition by CAFs can induce mechanical compression, leading to cytoplasmic exclusion of YAP in neighboring tumor cells and thus suppressing their proliferation (*196*). This duality underscores the complexity of Hippo pathway regulation in disease, posing both challenges and opportunities for therapeutic targeting of YAP/TAZ-TEAD interactions (*189*). Recent studies have also uncovered roles for YAP in immune regulation, particularly in macrophages. Substrate stiffness dynamically controls YAP nuclear localization and activity, modulating the macrophage inflammatory response to stimuli such as lipopolysaccharide. Macrophages cultured on soft ECMs show cytoplasmic YAP retention, reduced proinflammatory cytokine production, and increased interleukin-10 expression compared to those on stiffer substrates (*197*).

Together, these insights reveal the dual nature of YAP/TAZ in medicine: They are essential for tissue regeneration yet can also drive pathological remodeling and cancer progression. Their activity, finely tuned by nuclear mechanics, matrix viscoelastic properties, and cytoskeletal forces, represents a promising therapeutic axis for next-generation mechanomedicine.

### ERK signaling is a mechanically tuned pathway in disease progression

The ERK pathway acts as a crucial node linking mechanical signals to disease pathogenesis. In diabetic wound healing, magneto-induced dynamic mechanical stimulation of fibroblasts enhances keratinocyte activity via ERK, promoting angiogenesis and wound closure (*198*). In organ development, ERK activation in response to tissue curvature promotes actin polymerization and drives morphogenetic events. Mutations in the ERK pathway further emphasize its importance in pathological scenarios. For example, V-Ki-ras2 Kirsten rat sarcoma viral oncogene homolog (KRAS) or B-Raf proto-oncogene, serine/threonine kinase (BRAF) mutations—observed in 50 to 80% of melanomas—alter ERK pulses dynamics, driving oncogenic phenotypes (*199*, *200*). These mutations often disrupt negative feedback loops, preventing proper inhibition of pathway kinases (*201*, *202*). In uveal melanoma (Fig. 6C), the most common form of eye cancer, BRAF is frequently mutated, promoting uncontrolled cell growth and tumor progression (Fig. 6Ca) (*203*). In vitro studies demonstrate that ERK maintains a sustained activity and a nucleus concentration higher than in the cytoplasm (Fig. 6C, b and c), leading to a pathological cell fate, when MAPK pathway proteins are mutated. Recent phase 2 clinical trials have tested competitive ERK inhibitors in patients with this aggressive and treatment-resistant cancer (*204*–*206*), highlighting ERK as a promising therapeutic target. Ulixertinib (BVD-523) is an ATP-competitive inhibitor of ERK1/2 causing a conformational change and altering the principal kinases function of ERK proteins proving to be a potential anticancer treatment through inhibition of the MAPK pathway. Moving forward, developing less invasive and more localized therapeutic approaches that leverage ERK’s mechanosensitivity may offer more tissue-specific and less toxic interventions in oncology and regenerative medicine.

Across diverse diseases—spanning cancer, cardiovascular disease, fibrosis, immune disorders, and neurodegeneration—mechanical forces emerge not as passive by-products of disease but as active drivers of pathogenesis. These forces regulate cellular behavior, activate key signaling pathways such as YAP/TAZ and ERK, and modulate ECM remodeling. To translate this knowledge into effective therapies, mechanistically targeted interventions that restore or redirect mechanical signaling must be developed, whether through biomaterials, pathway inhibitors, or microenvironment engineering. As mechanomedicine evolves, integrating mechanobiological principles will be essential for designing innovative, context-specific treatments that address the fundamental mechanical underpinnings of disease.

## HYDROGELS AND MECHANOBIOLOGY IN REGENERATIVE MEDICINE

Hydrogels have emerged as a versatile class of biomaterials in regenerative medicine, offering tunable mechanical, chemical, and biological properties that can mimic the native ECM and modulate cellular behavior (*207*). Recent advances have leveraged hydrogel platforms not only for in vitro modeling but also for therapeutic applications, where their mechanical properties—particularly stiffness, viscoelasticity, and degradability—play a critical role in guiding cell fate decisions. Below, we highlight key developments in hydrogel design that illustrate how the mechanobiology of these materials is being harnessed for regenerative medicine.

### ECM viscoelasticity and cancer therapy

ECM viscoelasticity has emerged as a critical factor in regulating tumor progression and shaping the immune microenvironment. In cancer, altered ECM viscoelasticity not only drives malignancy but also influences the efficacy of immunotherapies, presenting an opportunity for therapeutic intervention (*208*, *209*). Recent advances have harnessed this concept by developing viscoelastic hydrogels that better mimic the native ECM, enabling both physiologically relevant in vitro models and mechanical modulation of immune cells for translational applications.

**Fig. 7.**
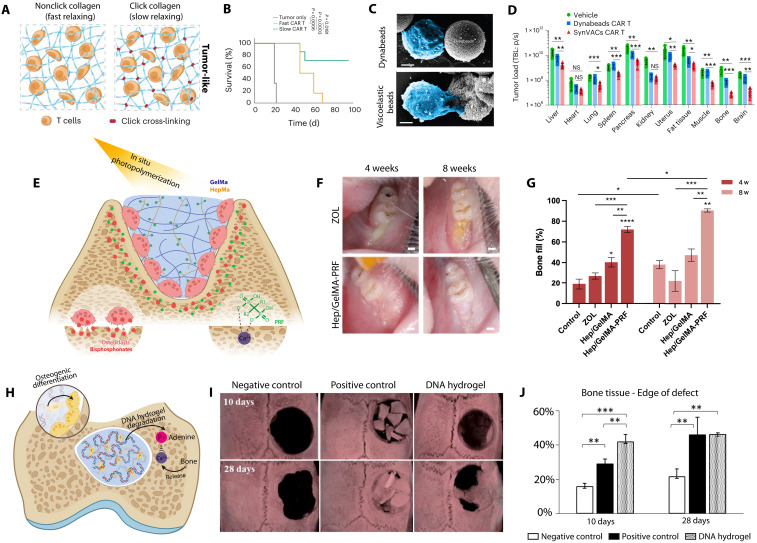
Hydrogels and mechanobiology in regenerative medicine. (**A** and **B**) Modulating T cell function. (A) Schematic of collagen I ECMs with tunable stress relaxation, designed to mimic healthy and cancerous microenvironments and modulate T cell immune properties. (B) In a mouse lymphoma model, CAR T cells cultured in tumor-like, slow-relaxing hydrogels (slow CAR T) improved survival compared to fast-relaxing hydrogel CAR T (fast CAR T). d, days. (A) and (B) Adapted from (*208*). (**C** and **D**) Viscoelastic microgels for CAR T activation. (C) Scanning electron microscopy image of T cells (blue) interacting with rigid elastic beads (Dynabeads; top) and viscoelastic beads [synthetic viscoelastic antigen-presenting cells (SynVACs); bottom] after 1 day of culture (both gray). Scale bars, 2 μm. (D) Quantification of tumor bioluminescence (TBL) signal in ovarian tumor xenograft mice. SynVAC-activated CAR T cells reduced tumor load in metastatic organs compared to vehicle or Dynabeads CAR T. NS, not significant. (C) and (D) Adapted from (*209*). (**E** to **G**) Hydrogel therapies for bone regeneration and osteonecrosis. (E) Schematic of a methacrylated heparin (HepMA)/gelatin methacryloyl (GelMA)–platelet-rich fibrin (PRF) hydrogel polymerized with UV light in the extraction fossa. Bisphosphonates (BPs) contribute to osteonecrosis by binding hydroxyapatite (HAP) crystals in the bone via two adjacent phosphonic acid groups chelating calcium, forming a strong bond. Created in BioRender, S.G. (2025) (https://BioRender.com/l2tneqs). (F) Posttreatment outcomes after 4 and 8 weeks with ZOL and Hep/GelMA-PRF hydrogels. Scale bars, 1 mm. (G) Graph of bone filling percentages for control, ZOL, Hep/GelMA hydrogel, and Hep/GelMA-PRF groups. w, weeks. (F) and (G) adapted from (*221*). (**H** to **J**) DNA hydrogels for calvarial defects. (H) Schematic of DNA hydrogel promoting bone regeneration. The hydrogel degrades physiologically, releasing phosphate ions and adenine to support osteogenic differentiation. Created in BioRender, S.G. (2025) (https://BioRender.com/l2tneqs). (I) Micro-CT images of rat calvarial defects at 10 and 28 days postsurgery for buffer (negative control), autogenous graft (positive control), and DNA hydrogel. (J) Quantification of bone tissue at defect edges in each group. (I) and (J) Adapted from (*226*). *P ≤ 0.05; **P ≤ 0.01; ***P ≤ 0.001; ****P ≤ 0.0001.

For example, engineered viscoelastic collagen I hydrogels have been shown to modulate the functional properties of T lymphocytes, including genetically modified subsets such as chimeric antigen receptor (CAR) T cells (*208*). In particular, ECMs with slower stress relaxation enhanced the cytotoxic activity of CAR T cells by up-regulating the activating protein 1 signaling pathway, an effect confirmed in vivo using a human lymphoma mouse model (Fig. 7A) (*208*).

Building on this, high-throughput microfluidic techniques have enabled the fabrication of synthetic viscoelastic antigen-presenting cells (SynVACs) composed of alginate-based microgels functionalized with anti-CD3 and anti-CD28 antibodies (*137*). These tunable platforms promote CAR T cell expansion under fast-relaxing conditions, resulting in superior antitumor activity and improved survival in vivo (Fig. 7B). SynVAC-generated CAR T cells also outperformed those produced using Dynabeads, the current clinical standard, in both in vitro tumors killing (Fig. 7C) and in vivo efficacy across multiple tumor models and sites (Fig. 7D).

Together, these studies position viscoelastic hydrogels as powerful tools to enhance CAR T cell therapies in cancer treatment. By replicating the dynamic mechanical environment of the ECM, they offer a promising route to improve immune cell function, increase therapeutic efficacy, and address current challenges in adoptive cell therapies (*208*, *209*).

### Photocrosslinked composite hydrogels for tissue regeneration

Photocrosslinkable composite hydrogels represent a major advancement in tissue engineering and regenerative medicine, offering precise control over critical material properties. Photochemical cross-linking enables the creation of stable, biomimetic scaffolds that replicate key features of the ECM and support essential cellular functions including proliferation, differentiation, and remodeling, making them versatile platforms for regenerative applications (*210*).

Natural polymers such as chitosan, gelatin methacryloyl (GelMA), and hyaluronic acid have been widely used because of their biocompatibility, tunability, and ease of functionalization. These materials have found applications in mechanobiology, regenerative medicine, cell transplantation, disease modeling, and drug delivery (*211*–*214*), driving the development of increasingly sophisticated materials for tissue repair and regeneration.

A compelling application of composite hydrogels lies in addressing the challenges associated with bisphosphonate (BP) therapies (*215*). While drugs such as zoledronic acid (ZOL), a potent inhibitor of bone resorption used in the treatment of osteoporosis and cancer-related bone conditions, effectively treat osteoporosis and cancer-related bone loss, long-term use can lead to medication-related osteonecrosis of the jaw (MRONJ), which is a severe condition linked to impaired bone turnover (*216*). BPs strongly bind hydroxyapatite (HAP) crystals in bone via bi- or tridentate chelation and, once internalized by osteoclasts, inhibit ATP synthesis and the mevalonate pathway, disrupting osteoclast function and triggering apoptosis (*217*). This results in impaired osteoclast function, reduced bone resorption, and, ultimately, osteoclast apoptosis. However, excessive inhibition of bone turnover can compromise normal tissue repair, predisposing patients to MRONJ.

To address this, innovative hydrogel-based strategies have been developed (*218*–*220*). Among these, biomaterial-based therapeutic design based on a composite hydrogel comprising GelMA, methacrylated heparin (HepMA), and platelet-rich fibrin (PRF) has shown strong therapeutic potential. This system, delivered as a liquid and photocrosslinked in situ, creates a stable scaffold that releases PRF-derived growth factors directly at the wound site (Fig. 7E). In preclinical models of BP-related osteonecrosis, Hep/GelMA-PRF hydrogels significantly enhanced bone regeneration compared to ZOL-treated controls, which exhibited delayed healing, chronic inflammation, and extensive necrosis by week 8 (Fig. 7F) (*221*). The inclusion of PRF further reduced necrotic area relative to Hep/GelMA alone, highlighting its synergistic role in tissue repair. Quantitative analyses confirmed the highest bone fill in the Hep/GelMA-PRF group (Fig. 7G), underscoring the mechanobiological potential of this matrix to counteract pharmacologically induced healing defects (*221*).

Collectively, these findings underscore the transformative potential of photocrosslinked composite hydrogels in tissue engineering. By integrating mechanical tunability with bioactive molecule delivery, these materials provide a powerful platform for promoting tissue regeneration in challenging clinical scenarios. Looking forward, innovations, such as programmable, dynamic materials, including DNA-based hydrogels, are set to further expand their versatility and therapeutic compromise in regenerative medicine.

### DNA-based hydrogels in regenerative medicine

DNA-based hydrogels have emerged as a promising class of biomaterials, combining biocompatibility, biodegradability, and the programmability of synthetic systems. Their sequence-defined architecture, dynamic cross-linking, and tunable mechanical properties make them a versatile platform for regenerative medicine applications (*222*, *223*). By providing control over both mechanical and biochemical cues, DNA hydrogels offer scaffolds that mimic the native ECM and are suitable for clinical translation.

#### Bone regeneration

Recent studies have demonstrated the osteoconductive properties of DNA-based hydrogels, showing their ability to support osteogenic cell viability, matrix deposition, and HAP mineralization—the main inorganic component of bone (*224*–*226*). A key advantage lies in their degradability, which release phosphate ions and adenine, both essential for osteogenic differentiation. Phosphate ions contribute to calcium phosphate formation and HAP mineralization, while adenine supports cell migration and osteogenic gene expression (Fig. 7H). In rat calvarial defect models (*226*), DNA hydrogels promoted bone growth as early as 10 days postinjection and achieved substantial defect filling by 28 days (Fig. 7, I and J). Although early degradation was observed, the material effectively enhanced osteoblast activity and matched the healing levels of autologous bone grafts, outperforming buffer-treated controls.

#### Cartilage and neural tissue applications

Beyond bone repair, DNA hydrogels have shown potential in cartilage and neural tissue regeneration. In osteoarthritis models, they improved retention and viability of bone marrow–derived MSCs, leading to enhanced cartilage repair and regeneration in rabbits (*227*). In neural applications, highly porous DNA hydrogels supported neurogenesis and spinal cord recovery (*228*). Preclinical studies in mouse models of spinal cord injury (SCI) revealed that these hydrogels support the migration and differentiation of neural stem cells (NSCs) and facilitated neural network formation at injury sites, resulting in functional improvement. Together, these findings underscore the broad utility of DNA hydrogels as dynamic, programmable scaffolds for regenerative therapies across diverse tissue types.

### Therapies combining stem cells and hydrogels in regenerative medicine

The ability to control stem cell differentiation through the mechanical properties of the cellular microenvironment has become a cornerstone of mechanobiology and regenerative medicine. Since the foundational work of Discher *et al*. (*14*), Engler *et al*. (*229*), and Smith *et al*. (*230*), it is well established that stem cells are highly responsive to matrix mechanical cues, which can direct their fate and function.

Stem cells are powerful tool due to their capacity for self-renewal and differentiation into specialized cell types. They can be derived from reprogramming of somatic cells as iPSCs or harvested from adult niches, such as MSCs from the bone marrow or NSCs from the hippocampus. However, the therapeutic success of stem cell–based therapies critically depends on microenvironmental signals, underscoring the critical interplay between stem cells and their ECM.

Hydrogels offer precise control over these cues by modulating matrix stiffness, viscoelasticity, and biochemical composition. Studies have shown that softer hydrogels promote neurogenic differentiation, while stiffer ones guide osteogenic fate (*230*, *231*). In addition, matrix mechanics influence the efficiency of cellular iPSC reprogramming, although the underlying molecular mechanisms remain under investigation.

**Fig. 8.**
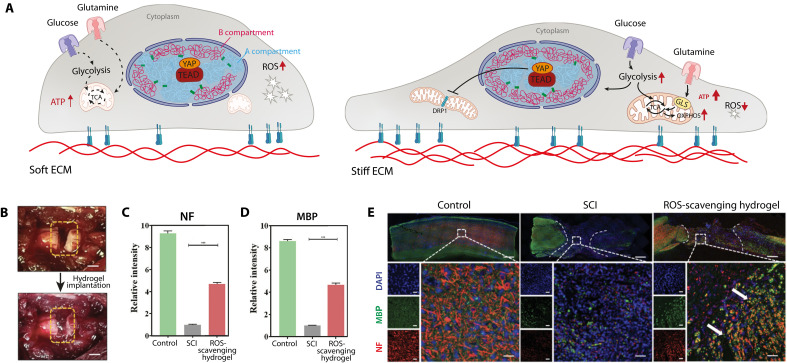
ECM stiffness and metabolic regulation in tissue repair. (**A**) Schematic of ECM stiffness–driven metabolism regulating osteogenic differentiation of MSCs via YAP. Stiff substrates enhance glycolysis (GLS) and oxidative phosphorylation (OXPHOS), supporting differentiation. DRP1 is dynamin-related protein 1, a GTPase that plays a crucial role in mitochondrial and peroxisomal fission. Created in BioRender, S.G. (2025) (https://BioRender.com/l2tneqs). Adapted from (*243*). (**B**) Photos of the spinal cord transection in rat model simulating SCI, treated with a reactive oxygen species (ROS)–scavenging hydrogel. (**C** and **D**) Quantitative analysis of neurofilament (NF; axon marker) and myelin basic protein (MBP; myelination marker) fluorescence intensity. (**E**) Immunostaining of nuclei (in blue), myelin basic protein (in green), and neurofilament (in red) around the spinal cord transection. White arrows indicate axons wrapped in myelin sheets arranged directionally. Scale bars, 100 μm. Adapted from (*233*). ***P ≤ 0.001.

Recent insights have revealed that substrate rigidity can directly influence the 3D organization of the genome, thereby regulating lineage-specific gene expression programs. Softer substrates, mimicking the mechanical properties of brain tissue, promote chromatin reorganization patterns that favor neural lineage commitment. In contrast, stiffer substrates resembling bone tissue induce chromatin condensation and gene expression profiles associated with osteogenic differentiation (Fig. 8A) (*232*). This mechanotransduction pathway, where mechanical signals are transmitted to the nucleus, inducing nuclear deformations and chromatin remodeling, provides a direct link between the physical properties of the ECM and the activation of lineage-specific transcriptional programs.

Many injuries involve ECM disruption, necessitating its replacement or supplementation with engineered hydrogels. When loaded with stem cells, these hydrogels not only replicate key physicochemical features of native ECM but also support tissue regeneration. However, hostile injury microenvironments, marked by inflammation and elevated reactive oxygen species (ROS), can impair stem cell function and differentiation. This is particularly problematic for SCI, where inflammatory niches pose major challenges for stem cell–based therapies.

ROS-scavenging hydrogels loaded with NSCs have been developed to address these barriers by modulating the pathological microenvironment. In a rat long-span transection SCI model, NSCs encapsulated within ROS-scavenging hydrogels promoted axonal regeneration and remyelination, as demonstrated by improved nerve tissue repair and motor function recovery (Fig. 8B). Axonal markers (Fig. 8C) and myelination markers (Fig. 8D) confirmed regeneration around the lesion site (Fig. 8E), underscoring the potential of NSC-laden ROS-scavenging hydrogels as an innovative strategy in regenerative medicine, particularly for SCI treatment (*233*). Together, these findings highlight the promise of combining stem cells with engineered hydrogels to create biomimetic microenvironments that guide cell fate, protect against hostile microenvironments, and enhance tissue regeneration across a wide range of regenerative medicine applications.

## TOWARD MECHANOMEDICINE: CHALLENGES AND FUTURE DIRECTIONS

Mechanobiology has emerged as a transformative frontier in the life sciences, revealing how physical forces and mechanical cues govern biological processes across scales—from molecular signaling and cellular behavior to tissue organization and organ function. These groundbreaking insights are now driving a new era of medical innovation, with profound implications for diagnostics, therapeutics, and regenerative medicine. While substantial progress has been made, particularly in regenerative applications, several nascent areas hold immense promise for future clinical translation.

One exciting direction is the interplay between mechanical forces and neural function. The mechanobiology of the nervous system is a rapidly evolving field with critical relevance to both neural repair and neurotechnology. For example, mechanobiological mechanisms underpin the formation of gliosis, a key response to neural injury that directly affects the longevity and functionality of bioelectronic implants (*234*). Deciphering how mechanical cues drive gliosis could inform the design of next-generation bioelectronic interfaces, reducing device failure and promoting neural repair. Similarly, mechanical forces are increasingly recognized as key regulators of neural development, guiding processes such as cell migration and differentiation. Amoeboid-like migration has been shown to play a pivotal role in retinal layer formation during vertebrate development (*235*), suggesting that targeted mechanical modulation could offer therapeutic strategies for neurodevelopmental disorders (*236*).

Mechanobiology is also reshaping the landscape of ocular medicine. The mechanical properties of the cornea are critical for maintaining visual acuity and tissue integrity, and altered corneal biomechanics are now recognized as a major contributor to diseases such as keratoconus (*237*). These insights are catalyzing the development of biomechanically informed interventions—including advanced tissue engineering strategies and laser-based procedures—that aim to restore or enhance corneal function. Beyond the eye, similar principles are being applied to other ECM-rich tissues, such as the vocal folds. By targeting the mechanophenotype of vocal fold tissues affected by cancer, it is possible to restore normal ECM mechanics and recalibrate cellular mechanosignaling, effectively reversing tumor progression and promoting tissue normalization (*238*). These approaches exemplify how mechanobiology can inform therapies that both inhibit disease and restore functional tissue architecture.

An emerging and particularly innovative application of mechanobiology lies in addressing the biological hallmarks of aging. Cellular senescence, a key driver of tissue degeneration, is profoundly influenced by mechanical forces. Recent studies demonstrate that applying specific mechanical cues to MSCs derived from aged donors can reduce senescence, enhancing their proliferation, multipotency, and regenerative capacity (*239*). These rejuvenating effects are mediated through the modulation of oxidative stress, activation of DNA repair pathways, and global transcriptomic reprogramming induced by mechanical conditioning (*240*). This research heralds a new frontier in antiaging therapies, offering strategies to delay age-related dysfunction, enhance tissue regeneration, and improve quality of life.

Looking ahead, several promising directions are poised to shape the future of mechanomedicine. Integrating mechanobiology with bioelectronics—for example, by developing bioelectronic interfaces that incorporate mechanosensitive elements—could revolutionize the design of neural prosthetics, wearable sensors, and diagnostic tools. Precision mechanomedicine, an emerging concept, envisions the development of personalized therapies tailored to patient-specific mechanical environments—such as the specific stiffness profiles of tumors or fibrotic tissues—offering an alternative paradigm for enhancing treatment efficacy. Last, the design of smart biomaterials that dynamically respond to mechanical cues holds immense promise for next-generation regenerative therapies, enabling the creation of adaptive scaffolds that evolve in concert with tissue healing processes.

However, a key translational challenge lies in the ubiquitous role of mechanical pathways in maintaining tissue homeostasis, raising concerns about potential off-target effects when therapeutically modulating these systems. To address this, future strategies may rely on spatially and temporally controlled delivery of mechanoactive agents, the development of cell type– or tissue-specific biomaterials, and the use of targeted bioelectronic systems that localize mechanical modulation to diseased microenvironments. By refining the precision of mechanical interventions, it will be possible to mitigate systemic effects while harnessing the full therapeutic potential of mechanobiology.

As mechanobiology continues to evolve, its integration into medical practice, what we might term mechanomedicine, offers the potential to revolutionize how tissues are diagnosed, treated, and regenerated across a wide range of pathologies. The coming decade will be critical for translating these advances from the laboratory to the clinic, marking the beginning of an era in which mechanical forces become central tools in precision medicine and regenerative therapies.

## References

[1] C. M. Nelson, B. Xiao, S. A. Wickström, Y. F. Dufrêne, D. J. Cosgrove, C.-P. Heisenberg, S. Dupont, A. E. Shyer, A. R. Rodrigues, X. Trepat, A. Diz-Muñoz, Mechanobiology: Shaping the future of cellular form and function. Cell 187, 2652–2656 (2024). 38788688 10.1016/j.cell.2024.04.006

[2] P. Roca-Cusachs, V. Conte, X. Trepat, Quantifying forces in cell biology. Nat. Cell Biol. 19, 742–751 (2017). 28628082 10.1038/ncb3564

[3] Y. Mao, S. A. Wickström, Mechanical state transitions in the regulation of tissue form and function. Nat. Rev. Mol. Cell Biol. 25, 654–670 (2024). 38600372 10.1038/s41580-024-00719-x

[4] X. Di, X. Gao, L. Peng, J. Ai, X. Jin, S. Qi, H. Li, K. Wang, D. Luo, Cellular mechanotransduction in health and diseases: From molecular mechanism to therapeutic targets. Signal Transduct. Target Ther. 8, 282 (2023). 37518181 10.1038/s41392-023-01501-9 PMC10387486

[5] K. Naruse, MECHANOMEDICINE: Applications of mechanobiology to medical sciences and next-generation medical technologies. J. Smooth Muscle Res. 54, 83–90 (2018). 30210090 10.1540/jsmr.54.83 PMC6135919

[6] K. Naruse, Mechanomedicine. Biophys. Rev. 10, 1257–1262 (2018). 30269290 10.1007/s12551-018-0459-7 PMC6233347

[7] D. Mohammed, M. Versaevel, C. Bruyère, L. Alaimo, M. Luciano, E. Vercruysse, A. Procès, S. Gabriele, Innovative tools for mechanobiology: Unraveling outside-in and inside-out mechanotransduction. Front. Bioeng. Biotechnol. 7, 162 (2019). 31380357 10.3389/fbioe.2019.00162 PMC6646473

[8] H. T. Ong, M. Sriram, H. H. Susapto, Y. Li, Y. Jiang, N. H. Voelcker, J. L. Young, A. W. Holle, R. Elnathan, The rise of mechanobiology for advanced cell engineering and manufacturing. Adv. Mater. 37, e2501640 (2025). 40576525 10.1002/adma.202501640 PMC12447053

[9] A. J. Engler, M. A. Griffin, S. Sen, C. G. Bönnemann, H. L. Sweeney, D. E. Discher, Myotubes differentiate optimally on substrates with tissue-like stiffness. J. Cell Biol. 166, 877–887 (2004). 15364962 10.1083/jcb.200405004 PMC2172122

[10] S. Treppo, H. Koepp, E. C. Quan, A. A. Cole, K. E. Kuettner, A. J. Grodzinsky, Comparison of biomechanical and biochemical properties of cartilage from human knee and ankle pairs. J. Orthop. Res. 18, 739–748 (2000). 11117295 10.1002/jor.1100180510

[11] P. C. Georges, J.-J. Hui, Z. Gombos, M. E. McCormick, A. Y. Wang, M. Uemura, R. Mick, P. A. Janmey, E. E. Furth, R. G. Wells, Increased stiffness of the rat liver precedes matrix deposition: Implications for fibrosis. Am. J. Physiol. Gastrointest. Liver Physiol. 293, G1147–G1154 (2007). 17932231 10.1152/ajpgi.00032.2007

[12] R. S. Nho, M. N. Ballinger, M. M. Rojas, S. N. Ghadiali, J. C. Horowitz, Biomechanical force and cellular stiffness in lung fibrosis. Am. J. Pathol. 192, 750–761 (2022). 35183510 10.1016/j.ajpath.2022.02.001 PMC9088200

[13] D. Sun, W. Gao, H. Hu, S. Zhou, Why 90% of clinical drug development fails and how to improve it? Acta Pharm. Sin. B 12, 3049–3062 (2022). 35865092 10.1016/j.apsb.2022.02.002 PMC9293739

[14] D. E. Discher, P. Janmey, Y. Wang, Tissue cells feel and respond to the stiffness of their substrate. Science 310, 1139–1143 (2005). 16293750 10.1126/science.1116995

[15] M. Luciano, S. Gabriele, Designing hydrogel dimensionality to investigate mechanobiology. Soft Matter 21, 4551–4572 (2025). 40439624 10.1039/d4sm01458h

[16] M. Chanduri, A. Kumar, D. Weiss, N. Emuna, I. Barsukov, M. Shi, K. Tanaka, X. Wang, A. Datye, J. Kanyo, F. Collin, T. Lam, U. D. Schwarz, S. Bai, T. Nottoli, B. T. Goult, J. D. Humphrey, M. A. Schwartz, Cellular stiffness sensing through talin 1 in tissue mechanical homeostasis. Sci. Adv. 10, eadi6286 (2024). 39167642 10.1126/sciadv.adi6286 PMC11338229

[17] N. Bansaccal, P. Vieugue, R. Sarate, Y. Song, E. Minguijon, Y. A. Miroshnikova, D. Zeuschner, A. Collin, J. Allard, D. Engelman, A.-L. Delaunois, M. Liagre, L. de Groote, E. Timmerman, D. Van Haver, F. Impens, I. Salmon, S. A. Wickström, A. Sifrim, C. Blanpain, The extracellular matrix dictates regional competence for tumour initiation. Nature 623, 828–835 (2023). 37968399 10.1038/s41586-023-06740-y PMC7615367

[18] A. Elosegui-Artola, A. Gupta, A. J. Najibi, B. Ri Seo, R. Garry, C. M. Tringides, I. de Lazaro, M. Darnell, W. Gu, Q. Zhou, D. A. Weitz, L. Manadevan, D. J. Mooney, Matrix viscoelasticity controls spatiotemporal tissue organization. Nat. Mater. 22, 117–127 (2023). 36456871 10.1038/s41563-022-01400-4 PMC10332325

[19] W. Fan, K. Adebowale, L. Váncza, Y. Li, M. F. Rabbi, K. Kunimoto, D. Chen, G. Mozes, D. K.-C. Chiu, Y. Li, J. Tao, Y. Wei, N. Adeniji, R. L. Brunsing, R. Dhanasekaran, A. Singhi, D. Geller, S. H. Lo, L. Hodgson, E. G. Engleman, G. W. Charville, V. Charu, S. P. Monga, T. Kim, R. G. Wells, O. Chaudhuri, N. J. Török, Matrix viscoelasticity promotes liver cancer progression in the pre-cirrhotic liver. Nature 626, 635–642 (2024). 38297127 10.1038/s41586-023-06991-9 PMC10866704

[20] M. Riaz, M. Versaevel, D. Mohammed, K. Glinel, S. Gabriele, Persistence of fan-shaped keratocytes is a matrix-rigidity-dependent mechanism that requires α5β1 integrin engagement. Sci. Rep. 6, 34141 (2016). 27678055 10.1038/srep34141 PMC5039689

[21] O. Chaudhuri, J. Cooper-White, P. A. Janmey, D. J. Mooney, V. B. Shenoy, Effects of extracellular matrix viscoelasticity on cellular behaviour. Nature 584, 535–546 (2020). 32848221 10.1038/s41586-020-2612-2 PMC7676152

[22] O. Chaudhuri, L. Gu, D. Klumpers, M. Darnell, S. A. Bencherif, J. C. Weaver, N. Huebsch, H. Lee, E. Lippens, G. N. Duda, D. J. Mooney, Hydrogels with tunable stress relaxation regulate stem cell fate and activity. Nat. Mater. 15, 326–334 (2016). 26618884 10.1038/nmat4489 PMC4767627

[23] S. Qiu, X. Zhao, J. Chen, J. Zeng, S. Chen, L. Chen, Y. Meng, B. Liu, H. Shan, M. Gao, Y. Feng, Characterizing viscoelastic properties of breast cancer tissue in a mouse model using indentation. J. Biomech. 69, 81–89 (2019). 10.1016/j.jbiomech.2018.01.007 29361276

[24] M. Cantini, H. Donnelly, M. J. Dalby, M. Salmeron-Sanchez, The plot thickens: The emerging role of matrix viscosity in cell mechanotransduction. Adv. Healthc. Mater. 9, e1901259 (2020). 31815372 10.1002/adhm.201901259

[25] Y. Ma, T. Han, Q. Yang, J. Wang, B. Feng, Y. Jia, Z. Wei, F. Xu, Viscoelastic cell microenvironment: Hydrogel-based strategy for recapitulating dynamic ECM mechanics. Adv. Funct. Mater. 31, 2100848 (2021).

[26] S. Nam, K. H. Hu, M. J. Butte, O. Chaudhuri, Strain-enhanced stress relaxation impacts nonlinear elasticity in collagen gels. Proc. Natl. Acad. Sci. U.S.A. 113, 5492–5497 (2016). 27140623 10.1073/pnas.1523906113 PMC4878492

[27] S. Nam, J. Lee, D. G. Brownfield, O. Chaudhuri, Viscoplasticity enables mechanical remodeling of matrix by cells. Biophys. J. 111, 2296–2308 (2016). 27851951 10.1016/j.bpj.2016.10.002 PMC5113260

[28] A. G. Clark, A. Maitra, C. Jacques, M. Bergert, C. Pérez-González, A. Simon, L. Lederer, A. Diz-Muñoz, X. Trepat, R. Voituriez, D. M. Vignjevic, Self-generated gradients steer collective migration on viscoelastic collagen networks. Nat. Mater. 21, 1200–1210 (2022). 35637338 10.1038/s41563-022-01259-5

[29] D. Sun, K. Zhang, F. Zheng, G. Yang, M. Yang, Y. Xu, Y. Qin, M. Lin, Y. Li, J. Tan, Q. Li, X. Qu, G. Li, L. Bian, C. Zhu, Matrix viscoelasticity controls differentiation of human blood vessel organoids into arterioles and promotes neovascularization in myocardial infarction. Adv. Mater. 37, e2410802 (2024). 39686788 10.1002/adma.202410802

[30] E. Barcelona-Estaje, M. A. G. Oliva, F. Cunniffe, A. Rodrigo-Navarro, P. Genever, M. J. Dalby, P. Roca-Cusachs, M. Cantini, M. Salmeron-Sanchez, N-cadherin crosstalk with integrin weakens the molecular clutch in response to surface viscosity. Nat. Commun. 15, 8824 (2024). 39394209 10.1038/s41467-024-53107-6 PMC11479646

[31] M. Bennett, M. Cantini, J. Reboud, J. M. Cooper, P. Roca-Cusachs, M. Salmeron-Sanchez, Molecular clutch drives cell response to surface viscosity. Proc. Natl. Acad. Sci. U.S.A. 115, 1192–1197 (2018). 29358406 10.1073/pnas.1710653115 PMC5819391

[32] G. Ciccone, M. Azevedo Gonzalez-Oliva, M. Versaevel, M. Cantini, M. Vassalli, M. Salmeron-Sanchez, S. Gabriele, Epithelial cell mechanoresponse to matrix viscoelasticity and confinement within micropatterned viscoelastic hydrogels. Adv. Sci. 12, e2408635 (2025). 10.1002/advs.202408635 PMC12079340 39950757

[33] T. Mitchison, M. Kirschner, Cytoskeletal dynamics and nerve growth. Neuron 1, 761–772 (1988). 3078414 10.1016/0896-6273(88)90124-9

[34] B. L. Bangasser, G. A. Shamsan, C. E. Chan, K. N. Opoku, E. Tüzel, B. W. Schlichtmann, J. A. Kasim, B. J. Fuller, B. R. McCullough, S. S. Rosenfeld, D. J. Odde, Shifting the optimal stiffness for cell migration. Nat. Commun. 8, 15313 (2017). 28530245 10.1038/ncomms15313 PMC5458120

[35] A. Elosegui-Artola, X. Trepat, P. Roca-Cusachs, Control of mechanotransduction by molecular clutch dynamics. Trends Cell Biol. 28, 356–367 (2018). 29496292 10.1016/j.tcb.2018.01.008

[36] Z. Gong, S. E. Szczesny, S. R. Caliari, E. E. Charrier, O. Chaudhuri, X. Cao, Y. Lin, R. L. Mauck, P. A. Janmey, J. A. Burdick, V. B. Shenoy, Matching material and cellular timescales maximizes cell spreading on viscoelastic substrates. Proc. Natl. Acad. Sci. U.S.A. 115, E2686–E2695 (2018). 29507238 10.1073/pnas.1716620115 PMC5866566

[37] K. Adebowale, Z. Gong, J. C. Hou, K. M. Wisdom, D. Garbett, H. Lee, S. Nam, T. Meyer, D. J. Odde, V. B. Shenoy, O. Chaudhuri, Enhanced substrate stress relaxation promotes filopodia-mediated cell migration. Nat. Mater. 20, 1290–1299 (2021). 33875851 10.1038/s41563-021-00981-w PMC8390443

[38] O. Courbot, A. Elosegui-Artola, The role of extracellular matrix viscoelasticity in development and disease. npj Biol. Phys. Mech. 2, 10 (2025). 40191103 10.1038/s44341-025-00014-6 PMC11968406

[39] M. Luciano, C. Tomba, A. Roux, S. Gabriele, How multiscale curvature couples forces to cellular functions. Nat. Rev. Phys. 6, 246–268 (2024).

[40] N. D. Bade, R. D. Kamien, R. K. Assoian, K. J. Stebe, Curvature and Rho activation differentially control the alignment of cells and stress fibers. Sci. Adv. 3, e1700150 (2017). 28913421 10.1126/sciadv.1700150 PMC5587136

[41] T. Chen, A. Callan-Jones, E. Fedorov, A. Ravasio, A. Brugués, H. T. Ong, Y. Toyama, B. C. Low, X. Trepat, T. Shemesh, R. Voituriez, B. Ladoux, Large-scale curvature sensing by directional actin flow drives cellular migration mode switching. Nat. Phys. 15, 393–402 (2019). 30984281 10.1038/s41567-018-0383-6 PMC6456019

[42] L. Pieuchot, J. Marteau, A. Guignandon, T. Dos Santos, I. Brigaud, P.-F. Chauvy, T. Cloatre, A. Ponche, T. Petithory, P. Rougerie, M. Vassaux, J.-L. Milan, N. Tusamda Wakhloo, A. Spangenberg, M. Bigerelle, K. Anselme, Curvotaxis directs cell migration through cell-scale curvature landscapes. Nat. Commun. 9, 3995 (2018). 30266986 10.1038/s41467-018-06494-6 PMC6162274

[43] R. K. Sadhu, M. Luciano, W. Xi, C. Martinez-Torres, M. Schröder, C. Blum, M. Tarantola, S. Villa, S. Penič, A. Iglič, C. Beta, O. Steinbock, E. Bodenschatz, B. Ladoux, S. Gabriele, N. S. Gov, A minimal physical model for curvotaxis driven by curved protein complexes at the cell’s leading edge. Proc. Natl. Acad. Sci. U.S.A. 121, e2306818121 (2024). 38489386 10.1073/pnas.2306818121 PMC10963004

[44] M. Luciano, M. Versaevel, Y. Kalukula, S. Gabriele, Mechanoresponse of curved epithelial monolayers lining bowl-shaped 3D microwells. Adv. Healthc. Mater. 13, e2203377 (2024). 37820698 10.1002/adhm.202203377

[45] S. A. Ruiz, C. S. Chen, Emergence of patterned stem cell differentiation within multicellular structures. Stem Cells 26, 2921–2927 (2008). 18703661 10.1634/stemcells.2008-0432 PMC2693100

[46] K. A. Kilian, B. Bugarija, B. T. Lahn, M. Mrksich, Geometric cues for directing the differentiation of mesenchymal stem cells. Proc. Natl. Acad. Sci. U.S.A. 107, 4872–4877 (2010). 20194780 10.1073/pnas.0903269107 PMC2841932

[47] D.-L. Pagès, E. Dornier, J. de Seze, E. Gontran, A. Maitra, A. Maciejewski, L. Wang, R. Luan, J. Cartry, C. Canet-Jourdan, J. Raingeaud, G. Lemahieu, M. Lebel, M. Ducreux, M. Gelli, J.-Y. Scoazec, M. Coppey, R. Voituriez, M. Piel, F. Jaulin, Cell clusters adopt a collective amoeboid mode of migration in confined nonadhesive environments. Sci. Adv. 8, eabp8416 (2022). 36179021 10.1126/sciadv.abp8416 PMC9524834

[48] D. Mohammed, G. Charras, E. Vercruysse, M. Versaevel, J. Lantoine, L. Alaimo, C. Bruyère, M. Luciano, K. Glinel, G. Delhaye, O. Théodoly, S. Gabriele, Substrate area confinement is a key determinant of cell velocity in collective migration. Nat. Phys. 15, 858–866 (2019).

[49] E. Vercruysse, D. B. Brückner, M. Gómez-González, A. Remson, M. Luciano, Y. Kalukula, L. Rossetti, X. Trepat, E. Hannezo, S. Gabriele, Geometry-driven migration efficiency of autonomous epithelial cell clusters. Nat. Phys. 20, 1492–1500 (2024).

[50] A. J. Lomakin, C. J. Cattin, D. Cuvelier, Z. Alraies, M. Molina, G. P. F. Nader, N. Srivastava, P. J. Sáez, J. M. Garcia-Arcos, I. Y. Zhitnyak, A. Bhargava, M. K. Driscoll, E. S. Welf, R. Fiolka, R. J. Petrie, N. S. De Silva, J. M. González-Granado, N. Manel, A. M. Lennon-Duménil, D. J. Müller, M. Piel, The nucleus acts as a ruler tailoring cell responses to spatial constraints. Science 370, eaba2894 (2020). 33060332 10.1126/science.aba2894 PMC8059074

[51] V. Venturini, F. Pezzano, F. Català Castro, H.-M. Häkkinen, S. Jiménez-Delgado, M. Colomer-Rosell, M. Marro, Q. Tolosa-Ramon, S. Paz-López, M. A. Valverde, J. Weghuber, P. Loza-Alvarez, M. Krieg, S. Wieser, V. Ruprecht, The nucleus measures shape changes for cellular proprioception to control dynamic cell behavior. Science 370, eaba2644 (2020). 33060331 10.1126/science.aba2644

[52] X. Gao, Y. Li, J. W. N. Lee, J. Zhou, V. Rangaraj, J. Marlena, A. W. Holle, Confined migration drives stem cell differentiation. Adv. Sci. 12, e2415407 (2025). 10.1002/advs.202415407 PMC12140319 40344472

[53] P. Friedl, P. B. Noble, P. A. Walton, D. W. Laird, P. J. Chauvin, R. J. Tabah, M. Black, K. S. Zänker, Migration of coordinated cell clusters in mesenchymal and epithelial cancer explants in vitro. Cancer Res. 55, 4557–4560 (1995). 7553628

[54] A. Carlsson, V. S. Nair, M. S. Luttgen, K. V. Keu, G. Horng, M. Vasanawala, A. Kolatkar, M. Jamali, A. H. Iagaru, W. Kuschner, B. W. Loo, J. B. Shrager, K. Bethel, C. K. Hoh, L. Bazhenova, J. Nieva, P. Kuhn, S. S. Gambhir, Circulating tumor microemboli diagnostics for patients with non–small-cell lung cancer. J. Thorac. Oncol. 9, 1111–1119 (2014). 25157764 10.1097/JTO.0000000000000235 PMC4145608

[55] K. J. Cheung, A. J. Ewald, A collective route to metastasis: Seeding by tumor cell clusters. Science 352, 167–169 (2016). 27124449 10.1126/science.aaf6546 PMC8183671

[56] B. L. Langille, Blood flow-induced remodeling of arteries in health and disease. Cardiovasc. Pathol. 1, 245–251 (1992). 25990420 10.1016/1054-8807(92)90034-L

[57] J. Ando, K. Yamamoto, Effects of shear stress and stretch on endothelial function. Antioxid. Redox Signal. 15, 1389–1403 (2011). 20854012 10.1089/ars.2010.3361

[58] A.-C. Vion, T. Perovic, C. Petit, I. Hollfinger, E. Bartels-Klein, E. Frampton, E. Gordon, L. Claesson-Welsh, H. Gerhardt, Endothelial cell orientation and polarity are controlled by shear stress and VEGF through distinct signaling pathways. Front. Physiol. 11, 623769 (2021). 33737879 10.3389/fphys.2020.623769 PMC7960671

[59] K. Moise, K. M. Arun, M. Pillai, J. Salvador, A. S. Mehta, Y. Goyal, M. L. Iruela-Arispe, Endothelial cell elongation and alignment in response to shear stress requires acetylation of microtubules. Front. Physiol. 15, 1425620 (2024). 39318362 10.3389/fphys.2024.1425620 PMC11420013

[60] S. X, C. Aitken, V. Mehta, B. Tardajos-Ayllon, J. Serbanovic-Canic, J. Zhu, B. Miao, E. Tzima, P. Evans, Y. Fang, M. A. Schwartz, Controversy in mechanotransduction - the role of endothelial cell-cell junctions in fluid shear stress sensing. J. Cell Sci. 137, jcs262348 (2024). 39143856 10.1242/jcs.262348 PMC11423816

[61] S. Huveneers, L.-K. Phng, Endothelial cell mechanics and dynamics in angiogenesis. Curr. Opin. Cell Biol. 91, 102441 (2024). 39342870 10.1016/j.ceb.2024.102441

[62] K. Sunderland, J. Jiang, F. Zhao, Disturbed flow’s impact on cellular changes indicative of vascular aneurysm initiation, expansion, and rupture: A pathological and methodological review. J. Cell. Physiol. 237, 278–300 (2022). 34486114 10.1002/jcp.30569 PMC8810685

[63] I. A. Tamargo, K. I. Baek, Y. Kim, C. Park, H. Jo, Flow-induced reprogramming of endothelial cells in atherosclerosis. Nat. Rev. Cardiol. 20, 738–753 (2023). 37225873 10.1038/s41569-023-00883-1 PMC10206587

[64] Y. Fang, D. Wu, K. G. Birukov, Mechanosensing and mechanoregulation of endothelial cell functions. Compr. Physiol. 9, 873–904 (2019). 30873580 10.1002/cphy.c180020 PMC6697421

[65] J. Z. Kechagia, J. Ivaska, P. Roca-Cusachs, Integrins as biomechanical sensors of the microenvironment. Nat. Rev. Mol. Cell Biol. 20, 457–473 (2019). 31182865 10.1038/s41580-019-0134-2

[66] I. Dasgupta, D. McCollum, Control of cellular responses to mechanical cues through YAP/TAZ regulation. J. Biol. Chem. 294, 17693–17706 (2019). 31594864 10.1074/jbc.REV119.007963 PMC6873206

[67] B. Cheng, W. Wan, G. Huang, Y. Li, G. M. Genin, M. R. K. Mofrad, T. J. Lu, F. Xu, M. Lin, Nanoscale integrin cluster dynamics controls cellular mechanosensing via FAKY397 phosphorylation. Sci. Adv. 6, eaax1909 (2020). 32181337 10.1126/sciadv.aax1909 PMC7056303

[68] L. B. Case, M. De Pasquale, L. Henry, M. K. Rosen, Synergistic phase separation of two pathways promotes integrin clustering and nascent adhesion formation. eLife 11, e72588 (2022). 35049497 10.7554/eLife.72588 PMC8791637

[69] B. Pontes, P. Monzo, L. Gole, A.-L. Le Roux, A. J. Kosmalska, Z. Y. Tam, W. Luo, S. Kan, V. Viasnoff, P. Roca-Cusachs, L. Tucker-Kellogg, N. C. Gauthier, Membrane tension controls adhesion positioning at the leading edge of cells. J. Cell Biol. 216, 2959–2977 (2017). 28687667 10.1083/jcb.201611117 PMC5584154

[70] T. Corne, T. Sieprath, J. Vandenbussche, D. Mohammed, M. te Lindert, K. Gevaert, S. Gabriele, K. Wolf, W. H. de Vos, Deregulation of focal adhesion formation and cytoskeletal tension due to loss of A-type lamins. Cell Adh. Migr. 11, 447–463 (2017). 27791462 10.1080/19336918.2016.1247144 PMC5810761

[71] S. Yang, X. Miao, S. Arnold, B. Li, A. T. Ly, H. Wang, M. Wang, X. Guo, M. M. Pathak, W. Zhao, C. D. Cox, Z. Shi, Membrane curvature governs the distribution of Piezo1 in live cells. Nat. Commun. 13, 7467 (2022). 36463216 10.1038/s41467-022-35034-6 PMC9719557

[72] H. J. Wang, Y. Wang, S. S. Mirjavadi, T. Andersen, L. Moldovan, P. Vatankhah, B. Russell, J. Jin, Z. Zhou, Q. Li, C. D. Cox, Q. P. Su, L. A. Ju, Microscale geometrical modulation of PIEZO1 mediated mechanosensing through cytoskeletal redistribution. Nat. Commun. 15, 5521 (2024). 38951553 10.1038/s41467-024-49833-6 PMC11217425

[73] M. A. G. Oliva, G. Ciccone, J. Luo, J. L. Voigt, P. Romani, O. Dobre, S. Dupont, M. Vassalli, M. Salmeron-Sanchez, Piezo1 is a mechanosensor of soft matrix viscoelasticity. bioRxiv 600570 [Preprint] (2024). 10.1101/2024.06.25.600570.

[74] Y. Liu, H. Tian, Y. Hu, Y. Cao, H. Song, S. Lan, Z. Dai, W. Chen, Y. Zhang, Z. Shao, Y. Liu, W. Tong, Mechanosensitive Piezo1 is crucial for periosteal stem cell-mediated fracture healing. Int. J. Biol. Sci. 18, 3961–3980 (2022). 35844802 10.7150/ijbs.71390 PMC9274506

[75] A. Totaro, T. Panciera, S. Piccolo, YAP/TAZ upstream signals and downstream responses. Nat. Cell Biol. 20, 888–899 (2018). 30050119 10.1038/s41556-018-0142-z PMC6186418

[76] T. Kawaue, I. Yow, Y. Pan, A. P. Le, Y. Lou, M. Loberas, M. Shagirov, X. Teng, J. Prost, T. Hiraiwa, B. Ladoux, Y. Toyama, Inhomogeneous mechanotransduction defines the spatial pattern of apoptosis-induced compensatory proliferation. Dev. Cell 58, 267–277.e5 (2023). 36800994 10.1016/j.devcel.2023.01.005 PMC7614677

[77] A. Sousa-Ortega, J. Vázquez-Marín, E. Sanabria-Reinoso, J. Corbacho, R. Polvillo, A. Campoy-López, L. Buono, F. Loosli, M. Almuedo-Castillo, J. R. Martínez-Morales, A Yap-dependent mechanoregulatory program sustains cell migration for embryo axis assembly. Nat. Commun. 14, 2804 (2023). 37193708 10.1038/s41467-023-38482-w PMC10188487

[78] S. R. Shah, C. Ren, N. D. Tippens, J. Park, A. Mohyeldin, S. Wang, G. Vela, J. C. Martinez-Gutierrez, S. S. Margolis, S. Schmidt, A. Quiñones-Hinojosa, A. Levchenko, YAP controls cell migration and invasion through a Rho GTPase switch. Sci. Signal. 18, eadu3794 (2025). 40424361 10.1126/scisignal.adu3794 PMC12268267

[79] C. Bruyère, M. Versaevel, D. Mohammed, L. Alaimo, M. Luciano, E. Vercruysse, S. Gabriele, Actomyosin contractility scales with myoblast elongation and enhances differentiation through YAP nuclear export. Sci. Rep. 9, 15565 (2019). 31664178 10.1038/s41598-019-52129-1 PMC6820726

[80] K. Meyer, N. C. Lammers, L. J. Bugaj, H. G. Garcia, O. D. Weiner, Optogenetic control of YAP reveals a dynamic communication code for stem cell fate and proliferation. Nat. Commun. 14, 6929 (2023). 37903793 10.1038/s41467-023-42643-2 PMC10616176

[81] M. Luciano, S.-L. Xue, W. H. De Vos, L. Redondo-Morata, M. Surin, F. Lafont, E. Hannezo, S. Gabriele, Cell monolayers sense curvature by exploiting active mechanics and nuclear mechanoadaptation. Nat. Phys. 17, 1382–1390 (2021).

[82] C. Huerta-López, A. Clemente-Manteca, D. Velázquez-Carreras, F. M. Espinosa, J. G. Sanchez, Á. Martínez-del-Pozo, M. García-García, S. Martín-Colomo, A. Rodríguez-Blanco, R. Esteban-González, F. M. Martín-Zamora, L. I. Gutierrez-Rus, R. Garcia, P. Roca-Cusachs, A. Elosegui-Artola, M. A. del Pozo, E. Herrero-Galán, P. Sáez, G. R. Plaza, J. Alegre-Cebollada, Cell response to extracellular matrix viscous energy dissipation outweighs high-rigidity sensing. Sci. Adv. 10, eadf9758 (2024). 39546608 10.1126/sciadv.adf9758 PMC11567001

[83] K. Aoki, Y. Kondo, H. Naoki, T. Hiratsuka, R. E. Itoh, M. Matsuda, Propagating wave of ERK activation orients collective cell migration. Dev. Cell 43, 305–317.e5 (2017). 29112851 10.1016/j.devcel.2017.10.016

[84] P. A. Gagliardi, M. Dobrzyński, M.-A. Jacques, C. Dessauges, P. Ender, Y. Blum, R. M. Hughes, A. R. Cohen, O. Pertz, Collective ERK/Akt activity waves orchestrate epithelial homeostasis by driving apoptosis-induced survival. Dev. Cell 56, 1712–1726.e6 (2021). 34081908 10.1016/j.devcel.2021.05.007

[85] T. Hiratsuka, I. Bordeu, G. Pruessner, F. M. Watt, Regulation of ERK basal and pulsatile activity control proliferation and exit from the stem cell compartment in mammalian epidermis. Proc. Natl. Acad. Sci. U.S.A. 117, 17796–17807 (2020). 32651268 10.1073/pnas.2006965117 PMC7395546

[86] N. Hino, L. Rossetti, A. Marín-Llauradó, K. Aoki, X. Trepat, M. Matsuda, T. Hirashima, ERK-mediated mechanochemical waves direct collective cell polarization. Dev. Cell 53, 646–660.e8 (2020). 32497487 10.1016/j.devcel.2020.05.011

[87] P. E. Farahani, S. B. Lemke, E. Dine, G. Uribe, J. E. Toettcher, C. M. Nelson, Substratum stiffness regulates Erk signaling dynamics through receptor-level control. Cell Rep. 37, 110181 (2021). 34965432 10.1016/j.celrep.2021.110181 PMC8756379

[88] Y. Gao, J. Zhou, Z. Xie, J. Wang, C. Ho, Y. Zhang, Q. Li, Mechanical strain promotes skin fibrosis through LRG-1 induction mediated by ELK1 and ERK signalling. Commun. Biol. 2, 359 (2019). 31602408 10.1038/s42003-019-0600-6 PMC6778114

[89] Y. Hao, S. Cheng, Y. Tanaka, Y. Hosokawa, Y. Yalikun, M. Li, Mechanical properties of single cells: Measurement methods and applications. Biotechnol. Adv. 45, 107648 (2020). 33080313 10.1016/j.biotechadv.2020.107648

[90] T. L. Nguyen, E. R. Polanco, A. N. Patananan, T. A. Zangle, M. A. Teitell, Cell viscoelasticity is linked to fluctuations in cell biomass distributions. Sci. Rep. 10, 7403 (2020). 32366921 10.1038/s41598-020-64259-y PMC7198624

[91] P. Bouvet, C. Bevilacqua, Y. Ambekar, G. Antonacci, J. Au, S. Caponi, S. Chagnon-Lessard, J. Czarske, T. Dehoux, D. Fioretto, Y. Fu, J. Guck, T. Hamann, D. Heinemann, T. Jähnke, H. Jean-Ruel, I. Kabakova, K. Koski, N. Koukourakis, D. Krause, S. La Cavera, T. Landes, J. Li, H. Mahmodi, J. Margueritat, M. Mattarelli, M. Monaghan, D. R. Overby, F. Perez-Cota, E. Pontecorvo, R. Prevedel, G. Ruocco, J. Sandercock, G. Scarcelli, F. Scarponi, C. Testi, P. Török, L. Vovard, W. J. Weninger, V. Yakovlev, S.-H. Yun, J. Zhang, F. Palombo, A. Bilenca, K. Elsayad, Consensus statement on Brillouin light scattering microscopy of biological materials. Nat. Photonics 19, 681–691 (2025).

[92] M. Dembo, Y.-L. Wang, Stresses at the cell-to-substrate interface during locomotion of fibroblasts. Biophys. J. 76, 2307–2316 (1999). 10096925 10.1016/S0006-3495(99)77386-8 PMC1300203

[93] W. Thielicke, R. Sonntag, Particle image velocimetry for MATLAB: Accuracy and enhanced algorithms in PIVlab. J. Open Res. Softw. 9, 12 (2021).

[94] N. Hersch, B. Wolters, G. Dreissen, R. Springer, N. Kirchgeßner, R. Merkel, B. Hoffmann, The constant beat: Cardiomyocytes adapt their forces by equal contraction upon environmental stiffening. Biol. Open 2, 351–361 (2013). 23519595 10.1242/bio.20133830 PMC3603417

[95] J. d’Alessandro, A. Barbier--Chebbah, V. Cellerin, O. Benichou, R. M. Mège, R. Voituriez, B. Ladoux, Cell migration guided by long-lived spatial memory. Nat. Commun. 12, 4118 (2021). 34226542 10.1038/s41467-021-24249-8 PMC8257581

[96] A. Elosegui-Artola, R. Oria, Y. Chen, A. Kosmalska, C. Pérez-González, N. Castro, C. Zhu, X. Trepat, P. Roca-Cusachs, Mechanical regulation of a molecular clutch defines force transmission and transduction in response to matrix rigidity. Nat. Cell Biol. 18, 540–548 (2016). 27065098 10.1038/ncb3336

[97] C. Franck, S. A. Maskarinec, D. A. Tirrell, G. Ravichandran, Three-dimensional traction force microscopy: A new tool for quantifying cell-matrix interactions. PLOS ONE 6, e17833 (2011). 21468318 10.1371/journal.pone.0017833 PMC3066163

[98] B. C. H. Cheung, R. J. Abbed, M. Wu, S. E. Leggett, 3D traction force microscopy in biological gels: From single cells to multicellular spheroids. Annu. Rev. Biomed. Eng. 26, 93–118 (2024). 38316064 10.1146/annurev-bioeng-103122-031130 PMC12914736

[99] A. Shapeti, J. Barrasa-Fano, A. R. Abdel Fattah, J. de Jong, J. A. Sanz-Herrera, M. Pezet, S. Assou, E. de Vet, S. A. Elahi, A. Ranga, E. Faurobert, H. Van Oosterwyck, Force-mediated recruitment and reprogramming of healthy endothelial cells drive vascular lesion growth. Nat. Commun. 15, 8660 (2024). 39370485 10.1038/s41467-024-52866-6 PMC11456588

[100] J. Rheinlaender, H. Wirbel, T. E. Schäffer, Spatial correlation of cell stiffness and traction forces in cancer cells measured with combined SICM and TFM. RSC Adv. 11, 13951–13956 (2021). 35423943 10.1039/d1ra01277k PMC8697701

[101] C. Mark, T. J. Grundy, P. L. Strissel, D. Böhringer, N. Grummel, R. Gerum, J. Steinwachs, C. C. Hack, M. W. Beckmann, M. Eckstein, R. Strick, G. M. O’Neill, B. Fabry, Collective forces of tumor spheroids in three-dimensional biopolymer networks. eLife 9, e51912 (2020). 32352379 10.7554/eLife.51912 PMC7192581

[102] G. M. Whitesides, E. Ostuni, S. Takayama, X. Jiang, D. E. Ingber, Soft lithography in biology and biochemistry. Annu. Rev. Biomed. Eng. 3, 335–373 (2001). 11447067 10.1146/annurev.bioeng.3.1.335

[103] M. Théry, Micropatterning as a tool to decipher cell morphogenesis and functions. J. Cell Sci. 123, 4201–4213 (2010). 21123618 10.1242/jcs.075150

[104] Q. Tseng, I. Wang, E. Duchemin-Pelletier, A. Azioune, N. Carpi, J. Gao, O. Filhol, M. Piel, M. Théry, M. Balland, A new micropatterning method of soft substrates reveals that different tumorigenic signals can promote or reduce cell contraction levels. Lab Chip 11, 2231 (2011). 21523273 10.1039/c0lc00641f

[105] P.-O. Strale, A. Azioune, G. Bugnicourt, Y. Lecomte, M. Chahid, V. Studer, Multiprotein printing by light-induced molecular adsorption. Adv. Mater. 28, 2024–2029 (2016). 26689426 10.1002/adma.201504154

[106] T. Grevesse, M. Versaevel, G. Circelli, S. Desprez, S. Gabriele, A simple route to functionalize polyacrylamide hydrogels for the independent tuning of mechanotransduction cues. Lab Chip 13, 777–780 (2013). 23334710 10.1039/c2lc41168g

[107] M. Versaevel, L. Alaimo, V. Seveau, M. Luciano, D. Mohammed, C. Bruyère, E. Vercruysse, O. Théodoly, S. Gabriele, Collective migration during a gap closure in a two-dimensional haptotactic model. Sci. Rep. 11, 5811 (2021). 33712641 10.1038/s41598-021-84998-w PMC7954790

[108] K. V. Lane, L. P. Dow, E. A. Castillo, R. Boros, S. D. Feinstein, G. Pardon, B. L. Pruitt, Cell architecture and dynamics of human induced pluripotent stem cell-derived cardiomyocytes (hiPSC-CMs) on hydrogels with spatially patterned laminin and N-cadherin. ACS Appl. Mater. Interfaces 17, 174–186 (2025). 39680735 10.1021/acsami.4c11934 PMC11783353

[109] M. Luciano, M. Versaevel, E. Vercruysse, A. Procès, Y. Kalukula, A. Remson, A. Deridoux, S. Gabriele, Appreciating the role of cell shape changes in the mechanobiology of epithelial tissues. Biophys. Rev. 3, 011305 (2022). 10.1063/5.0074317 PMC10903419 38505223

[110] M. Versaevel, T. Grevesse, S. Gabriele, Spatial coordination between cell and nuclear shape within micropatterned endothelial cells. Nat. Commun. 3, 671 (2012). 22334074 10.1038/ncomms1668

[111] A. J. Jimenez, A. Schaeffer, C. De Pascalis, G. Letort, B. Vianay, M. Bornens, M. Piel, L. Blanchoin, M. Théry, Acto-myosin network geometry defines centrosome position. Curr. Biol. 31, 1206–1220.e5 (2021). 33609453 10.1016/j.cub.2021.01.002

[112] D. B. Brückner, A. Fink, C. Schreiber, P. J. F. Röttgermann, J. O. Rädler, C. P. Broedersz, Stochastic nonlinear dynamics of confined cell migration in two-state systems. Nat. Phys. 15, 595–601 (2019).

[113] Y. Kalukula, M. Luciano, G. Simanov, G. Charras, D. B. Brückner, S. Gabriele, The actin cortex acts as a mechanical memory of morphology in confined migrating cells. Nat. Phys. 21, 1451–1461 (2025).

[114] G. Sandu, J. A. Osses, M. Luciano, D. Caina, A. Stopin, D. Bonifazi, J.-F. Gohy, A. Silhanek, I. Florea, M. Bahri, O. Ersen, P. Leclère, S. Gabriele, A. Vlad, S. Melinte, Kinked silicon nanowires: Superstructures by metal-assisted chemical etching. Nano Lett. 19, 7681–7690 (2019). 31593477 10.1021/acs.nanolett.9b02568

[115] K. Hennig, I. Wang, P. Moreau, L. Valon, S. Debeco, M. Coppey, Y. A. Miroshnikova, C. Albiges-Rizo, C. Favard, R. Voituriez, M. Balland, Stick-slip dynamics of cell adhesion triggers spontaneous symmetry breaking and directional migration of mesenchymal cells on one-dimensional lines. Sci. Adv. 6, 5670 (2020). 10.1126/sciadv.aau5670 PMC6941913 31921998

[116] U. S. Schwarz, M. L. Gardel, United we stand: Integrating the actin cytoskeleton and cell-matrix adhesions in cellular mechanotransduction. J. Cell Sci. 125, 3051–3060 (2012). 22797913 10.1242/jcs.093716 PMC3434863

[117] E. Ng, A. Gopal, K. Hoshino, X. Zhang, Multicolor microcontact printing of proteins on nanoporous surface for patterned immunoassay. Appl. Nanosci. 1, 79–85 (2011).

[118] D. Chitsaz, T. E. Kennedy, High-throughput microcontact printing of proteins in microwell cell culture plates. MethodsX 12, 102665 (2024). 38524307 10.1016/j.mex.2024.102665 PMC10957495

[119] Q. Smith, E. Stukalin, S. Kusuma, S. Gerecht, S. X. Sun, Stochasticity and spatial interaction govern stem cell differentiation dynamics. Sci. Rep. 5, 12617 (2015). 26227093 10.1038/srep12617 PMC4521170

[120] S. M. Morgani, J. J. Metzger, J. Nichols, E. D. Siggia, A.-K. Hadjantonakis, Micropattern differentiation of mouse pluripotent stem cells recapitulates embryo regionalized cell fate patterning. eLife 7, e32839 (2018). 29412136 10.7554/eLife.32839 PMC5807051

[121] M. I. Maremonti, V. Panzetta, D. Dannhauser, P. A. Netti, F. Causa, Wide-range viscoelastic compression forces in microfluidics to probe cell-dependent nuclear structural and mechanobiological responses. J. R. Soc. Interface 19, 20210880 (2022). 35440204 10.1098/rsif.2021.0880 PMC9019521

[122] P. M. Davidson, G. R. Fedorchak, S. Mondésert-Deveraux, E. S. Bell, P. Isermann, D. Aubry, R. Allena, J. Lammerding, High-throughput microfluidic micropipette aspiration device to probe time-scale dependent nuclear mechanics in intact cells. Lab Chip 19, 3652–3663 (2019). 31559980 10.1039/c9lc00444k PMC6810812

[123] S. Gabriele, A.-M. Benoliel, P. Bongrand, O. Théodoly, Microfluidic investigation reveals distinct roles for actin cytoskeleton and myosin II activity in capillary leukocyte trafficking. Biophys. J. 96, 4308–4318 (2009). 19450501 10.1016/j.bpj.2009.02.037 PMC2712202

[124] A. Procès, Y. A. Alpizar, S. Halliez, B. Brône, F. Saudou, L. Ris, S. Gabriele, Stretch-injury promotes microglia activation with enhanced phagocytic and synaptic stripping activities. Biomaterials 305, 122426 (2024). 38134473 10.1016/j.biomaterials.2023.122426

[125] H. Yu, D. Kang, M. Whang, T. Kim, J. Kim, A microfluidic model artery for studying the mechanobiology of endothelial cells. Adv. Healthc. Mater. 10, e2100508 (2021). 34297476 10.1002/adhm.202100508

[126] R. C. Boot, A. Van Der Net, C. Gogou, P. Mehta, D. H. Meijer, G. H. Koenderink, P. E. Boukany, Cell spheroid viscoelasticity is deformation-dependent. Sci. Rep. 14, 20013 (2024). 39198595 10.1038/s41598-024-70759-y PMC11358509

[127] D. Soteriou, M. Kubánková, C. Schweitzer, R. López-Posadas, R. Pradhan, O.-M. Thoma, A.-H. Györfi, A.-E. Matei, M. Waldner, J. H. W. Distler, S. Scheuermann, J. Langejürgen, M. Eckstein, R. Schneider-Stock, R. Atreya, M. F. Neurath, A. Hartmann, J. Guck, Rapid single-cell physical phenotyping of mechanically dissociated tissue biopsies. Nat. Biomed. Eng 7, 1392–1403 (2023). 37024677 10.1038/s41551-023-01015-3 PMC10651479

[128] C. M. Leung, P. de Haan, K. Ronaldson-Bouchard, G.-A. Kim, J. Ko, H. S. Rho, Z. Chen, P. Habibovic, N. L. Jeon, S. Takayama, M. L. Shuler, G. Vunjak-Novakovic, O. Frey, E. Verpoorte, Y.-C. Toh, A guide to the organ-on-a-chip. Nat. Rev. Methods Primers 2, 33 (2022).

[129] K. Ronaldson-Bouchard, D. Teles, K. Yeager, D. N. Tavakol, Y. Zhao, A. Chramiec, S. Tagore, M. Summers, S. Stylianos, M. Tamargo, B. M. Lee, S. P. Halligan, E. H. Abaci, Z. Guo, J. Jacków, A. Pappalardo, J. Shih, R. K. Soni, S. Sonar, C. German, A. M. Christiano, A. Califano, K. K. Hirschi, C. S. Chen, A. Przekwas, G. Vunjak-Novakovic, A multi-organ chip with matured tissue niches linked by vascular flow. Nat. Biomed. Eng. 6, 351–371 (2022). 35478225 10.1038/s41551-022-00882-6 PMC9250010

[130] Z. N. Coker, M. Troyanova-Wood, Z. A. Steelman, B. L. Ibey, J. N. Bixler, M. O. Scully, V. V. Yakovlev, Brillouin microscopy monitors rapid responses in subcellular compartments. PhotoniX 5, 9 (2024). 38618142 10.1186/s43074-024-00123-w PMC11006764

[131] C. Conrad, K. M. Gray, K. M. Stroka, I. Rizvi, G. Scarcelli, Mechanical characterization of 3D ovarian cancer nodules using brillouin confocal microscopy. Cell Mol. Bioeng. 12, 215–226 (2019). 31719911 10.1007/s12195-019-00570-7 PMC6816613

[132] B. A. Loveless, K. A. Moin, P. C. Hoopes, M. Moshirfar, The utilization of brillouin microscopy in corneal diagnostics: A systematic review. Cureus 16, e65769 (2024). 39211657 10.7759/cureus.65769 PMC11361473

[133] Y.-F. Zhou, High intensity focused ultrasound in clinical tumor ablation. World J. Clin. Oncol. 2, 8–27 (2011). 21603311 10.5306/wjco.v2.i1.8 PMC3095464

[134] J. Liu, Y.-G. Feng, C. Zhang, W.-Z. Chen, Advancements in high-intensity focused ultrasound for urological tumor research and application. Ann. Med. Surg. 87, 2014–2019 (2025). 10.1097/MS9.0000000000002832 PMC11981266 40212168

[135] K. R. Levental, H. Yu, L. Kass, J. N. Lakins, M. Egeblad, J. T. Erler, S. F. T. Fong, K. Csiszar, A. Giaccia, W. Weninger, M. Yamauchi, D. L. Gasser, V. M. Weaver, Matrix crosslinking forces tumor progression by enhancing integrin signaling. Cell 139, 891–906 (2009). 19931152 10.1016/j.cell.2009.10.027 PMC2788004

[136] K. M. Wisdom, K. Adebowale, J. Chang, J. Y. Lee, S. Nam, R. Desai, N. S. Rossen, M. Rafat, R. B. West, L. Hodgson, O. Chaudhuri, Matrix mechanical plasticity regulates cancer cell migration through confining microenvironments. Nat. Commun. 9, 4144 (2018). 30297715 10.1038/s41467-018-06641-z PMC6175826

[137] M. Yamauchi, M. Shiiba, Lysine hydroxylation and cross-linking of collagen. Methods Mol. Biol. 446, 95–108 (2008). 18373252 10.1007/978-1-60327-084-7_7

[138] B. J. Pfeiffer, C. L. Franklin, F. Hsieh, R. A. Bank, C. L. Phillips, Alpha 2(I) collagen deficient oim mice have altered biomechanical integrity, collagen content, and collagen crosslinking of their thoracic aorta. Matrix Biol. 24, 451–458 (2005). 16095890 10.1016/j.matbio.2005.07.001

[139] J. T. Erler, K. L. Bennewith, T. R. Cox, G. Lang, D. Bird, A. Koong, Q.-T. Le, A. J. Giaccia, Hypoxia-induced lysyl oxidase is a critical mediator of bone marrow cell recruitment to form the premetastatic niche. Cancer Cell 15, 35–44 (2009). 19111879 10.1016/j.ccr.2008.11.012 PMC3050620

[140] D. Wenqiang, A. Novin, Y. Liu, J. Afzal, Y. Suhail, S. Liu, N. R. Gavin, J. R. Jorgensen, C. M. Morosky, R. Figueroa, T. A. Schmidt, M. Sanders, M. A. Brewer, Kshitiz, Scar matrix drives Piezo1 mediated stromal inflammation leading to placenta accreta spectrum. Nat. Commun. 15, 8379 (2024). 39333481 10.1038/s41467-024-52351-0 PMC11436960

[141] I. Malenica, J. Adam, S. Corgnac, L. Mezquita, E. Auclin, I. Damei, L. Grynszpan, G. Gros, V. de Montpréville, D. Planchard, N. Théret, B. Besse, F. Mami-Chouaib, Integrin-α_V_-mediated activation of TGF-β regulates anti-tumour CD8 T cell immunity and response to PD-1 blockade. Nat. Commun. 12, 5209 (2021). 34471106 10.1038/s41467-021-25322-y PMC8410945

[142] D. E. Kohan, E. W. Inscho, D. Wesson, D. M. Pollock, Physiology of endothelin and the kidney. Compr. Physiol. 1, 883–919 (2011). 23737206 10.1002/cphy.c100039 PMC3940435

[143] A. E. John, R. H. Graves, K. T. Pun, G. Vitulli, E. J. Forty, P. F. Mercer, J. L. Morrell, J. W. Barrett, R. F. Rogers, M. Hafeji, L. I. Bibby, E. Gower, V. S. Morrison, Y. Man, J. A. Roper, J. C. Luckett, L. A. Borthwick, B. S. Barksby, R. A. Burgoyne, R. Barnes, J. Le, D. J. Flint, S. Pyne, A. Habgood, L. A. Organ, C. Joseph, R. C. Edwards-Pritchard, T. M. Maher, A. J. Fisher, N. S. Gudmann, D. J. Leeming, R. C. Chambers, P. T. Lukey, R. P. Marshall, S. J. F. Macdonald, R. G. Jenkins, R. J. Slack, Translational pharmacology of an inhaled small molecule αvβ6 integrin inhibitor for idiopathic pulmonary fibrosis. Nat. Commun. 11, 4659 (2020). 32938936 10.1038/s41467-020-18397-6 PMC7494911

[144] P. Sime, G. Jenkins, Goldilocks and the three trials: Clinical trials targeting the α_v_β_6_ integrin in idiopathic pulmonary fibrosis. Am. J. Respir. Crit. Care Med. 206, 1062–1063 (2022). 36018580 10.1164/rccm.202208-1579ED PMC9704830

[145] S. R. Rahman, J. A. Roper, J. I. Grove, G. P. Aithal, K. T. Pun, A. J. Bennett, Integrins as a drug target in liver fibrosis. Liver Int. 42, 507–521 (2022). 35048542 10.1111/liv.15157

[146] P. Kollmannsberger, C. M. Bidan, J. W. C. Dunlop, P. Fratzl, V. Vogel, Tensile forces drive a reversible fibroblast-to-myofibroblast transition during tissue growth in engineered clefts. Sci. Adv. 4, eaao4881 (2018). 29349300 10.1126/sciadv.aao4881 PMC5771696

[147] P. Zhu, N.-H. Tseng, T. Xie, N. Li, I. Fitts-Sprague, S. R. Peyton, Y. Sun, Biomechanical microenvironment regulates fusogenicity of breast cancer cells. ACS Biomater Sci. Eng. 5, 3817–3827 (2019). 33438422 10.1021/acsbiomaterials.8b00861 PMC9800072

[148] H. A. Messal, S. Alt, R. M. M. Ferreira, C. Gribben, V. M.-Y. Wang, C. G. Cotoi, G. Salbreux, A. Behrens, Tissue curvature and apicobasal mechanical tension imbalance instruct cancer morphogenesis. Nature 566, 126–130 (2019). 30700911 10.1038/s41586-019-0891-2 PMC7025886

[149] S. Nemec, J. Lam, J. Zhong, C. Heu, P. Timpson, Q. Li, J. Youkhana, G. Sharbeen, P. A. Phillips, K. A. Kilian, Interfacial curvature in confined coculture directs stromal cell activity with spatial corralling of pancreatic cancer cells. Adv. Biol. 5, e2000525 (2021). 10.1002/adbi.202000525 33754491

[150] J. Lee, A. A. Abdeen, K. L. Wycislo, T. M. Fan, K. A. Kilian, Interfacial geometry dictates cancer cell tumorigenicity. Nat. Mater. 15, 856–862 (2016). 27043781 10.1038/nmat4610

[151] J. Gao, C. Yang, J. Li, S. Liu, Z. Ao, D. Han, Interfacial curvature as a potential index for prognosis of colon adenocarcinoma. Adv. Biol. 5, e1900277 (2021). 10.1002/adbi.201900277 33729697

[152] M. Krause, F. W. Yang, M. te Lindert, P. Isermann, J. Schepens, R. J. A. Maas, C. Venkataraman, J. Lammerding, A. Madzvamuse, W. Hendriks, J. te Riet, K. Wolf, Cell migration through three-dimensional confining pores: Speed accelerations by deformation and recoil of the nucleus. Philos. Trans. R. Soc. Lond. B Biol. Sci. 374, 20180225 (2019). 31431171 10.1098/rstb.2018.0225 PMC6627020

[153] C.-R. Hsia, J. McAllister, O. Hasan, J. Judd, S. Lee, R. Agrawal, C.-Y. Chang, P. Soloway, J. Lammerding, Confined migration induces heterochromatin formation and alters chromatin accessibility. iScience 25, 104978 (2022). 36117991 10.1016/j.isci.2022.104978 PMC9474860

[154] Ozmosi | Ricolinostat Drug Profile; https://pryzm.ozmosi.com/product/1884.

[155] L. Santo, T. Hideshima, A. L. Kung, J.-C. Tseng, D. Tamang, M. Yang, M. Jarpe, J. H. van Duzer, R. Mazitschek, W. C. Ogier, D. Cirstea, S. Rodig, H. Eda, T. Scullen, M. Canavese, J. Bradner, K. C. Anderson, S. S. Jones, N. Raje, Preclinical activity, pharmacodynamic, and pharmacokinetic properties of a selective HDAC6 inhibitor, ACY-1215, in combination with bortezomib in multiple myeloma. Blood 119, 2579–2589 (2012). 22262760 10.1182/blood-2011-10-387365 PMC3337713

[156] M. L. Decaris, J. R. Schaub, C. Chen, J. Cha, G. G. Lee, M. Rexhepaj, S. S. Ho, V. Rao, M. M. Marlow, P. Kotak, E. H. Budi, L. Hooi, J. Wu, M. Fridlib, S. P. Martin, S. Huang, M. Chen, M. Muñoz, T. F. Hom, P. J. Wolters, T. J. Desai, F. Rock, K. Leftheris, D. J. Morgans, E.-I. Lepist, P. Andre, E. A. Lefebvre, S. M. Turner, Dual inhibition of α_v_β_6_ and α_v_β_1_ reduces fibrogenesis in lung tissue explants from patients with IPF. Respir. Res. 22, 265 (2021). 34666752 10.1186/s12931-021-01863-0 PMC8524858

[157] M. R. Zanotelli, A. Rahman-Zaman, J. A. VanderBurgh, P. V. Taufalele, A. Jain, D. Erickson, F. Bordeleau, C. A. Reinhart-King, Energetic costs regulated by cell mechanics and confinement are predictive of migration path during decision-making. Nat. Commun. 10, 4185 (2019). 31519914 10.1038/s41467-019-12155-z PMC6744572

[158] G. P. de Freitas Nader, S. Agüera-Gonzalez, F. Routet, M. Gratia, M. Maurin, V. Cancila, C. Cadart, A. Palamidessi, R. N. Ramos, M. S. Roman, M. Gentili, A. Yamada, A. Williart, C. Lodillinsky, E. Lagoutte, C. Villard, J.-L. Viovy, C. Tripodo, J. Galon, G. Scita, N. Manel, P. Chavrier, M. Piel, Compromised nuclear envelope integrity drives TREX1-dependent DNA damage and tumor cell invasion. Cell 184, 5230–5246.e22 (2021). 34551315 10.1016/j.cell.2021.08.035

[159] E. S. Bell, P. Shah, N. Zuela-Sopilniak, D. Kim, A.-A. Varlet, J. L. P. Morival, A. L. McGregor, P. Isermann, P. M. Davidson, J. J. Elacqua, J. N. Lakins, L. Vahdat, V. M. Weaver, M. B. Smolka, P. N. Span, J. Lammerding, Low lamin A levels enhance confined cell migration and metastatic capacity in breast cancer. Oncogene 41, 4211–4230 (2022). 35896617 10.1038/s41388-022-02420-9 PMC9925375

[160] C. Souilhol, J. Serbanovic-Canic, M. Fragiadaki, T. J. Chico, V. Ridger, H. Roddie, P. C. Evans, Endothelial responses to shear stress in atherosclerosis: A novel role for developmental genes. Nat. Rev. Cardiol. 17, 52–63 (2020). 31366922 10.1038/s41569-019-0239-5

[161] M. Zhou, Y. Yu, R. Chen, X. Liu, Y. Hu, Z. Ma, L. Gao, W. Jian, L. Wang, Wall shear stress and its role in atherosclerosis. Front. Cardiovasc. Med. 10, 1083547 (2023). 37077735 10.3389/fcvm.2023.1083547 PMC10106633

[162] C. A. Dessalles, C. Ramón-Lozano, A. Babataheri, A. I. Barakat, Luminal flow actuation generates coupled shear and strain in a microvessel-on-chip. Biofabrication 14, 015003 (2022). 10.1088/1758-5090/ac2baa 34592728

[163] C. Leclech, D. Gonzalez-Rodriguez, A. Villedieu, T. Lok, A.-M. Déplanche, A. I. Barakat, Topography-induced large-scale antiparallel collective migration in vascular endothelium. Nat. Commun. 13, 2797 (2022). 35589751 10.1038/s41467-022-30488-0 PMC9120158

[164] C. A. Dessalles, N. Cuny, A. Boutillon, P. F. Salipante, A. Babataheri, A. I. Barakat, G. Salbreux, Interplay of actin nematodynamics and anisotropic tension controls endothelial mechanics. Nat. Phys. 21, 999–1008 (2025). 40546251 10.1038/s41567-025-02847-3 PMC12176649

[165] C. Xue, K. Chen, Z. Gao, T. Bao, L. Dong, L. Zhao, X. Tong, X. Li, Common mechanisms underlying diabetic vascular complications: Focus on the interaction of metabolic disorders, immuno-inflammation, and endothelial dysfunction. Cell Commun. Signal 21, 298 (2023). 37904236 10.1186/s12964-022-01016-w PMC10614351

[166] E. Westein, A. D. Van Der Meer, M. J. E. Kuijpers, J.-P. Frimat, A. Van Den Berg, J. W. M. Heemskerk, Atherosclerotic geometries exacerbate pathological thrombus formation poststenosis in a von Willebrand factor-dependent manner. Proc. Natl. Acad. Sci. U.S.A. 110, 1357–1362 (2013). 23288905 10.1073/pnas.1209905110 PMC3557050

[167] Y. C. Zhao, P. Vatankhah, T. Goh, R. Michelis, K. Kyanian, Y. Zhang, Z. Li, L. A. Ju, Hemodynamic analysis for stenosis microfluidic model of thrombosis with refined computational fluid dynamics simulation. Sci. Rep. 11, 6875 (2021). 33767279 10.1038/s41598-021-86310-2 PMC7994556

[168] R. Maringanti, C. G. M. Van Dijk, E. M. Meijer, M. M. Brandt, M. Li, V. P. C. Tiggeloven, M. M. Krebber, I. Chrifi, D. J. Duncker, M. C. Verhaar, C. Cheng, Atherosclerosis on a chip: A 3-dimensional microfluidic model of early arterial events in human plaques. Arterioscler. Thromb. Vasc. Biol. 44, 2453–2472 (2024). 39297206 10.1161/ATVBAHA.124.321332

[169] H. Hong, C. Li, J. L. Gutiérrez-Chico, Z. Wang, J. Huang, M. Chu, T. Kubo, L. Chen, W. Wijns, S. Tu, Radial wall strain: A novel angiographic measure of plaque composition and vulnerability. EuroIntervention 18, 1001–1010 (2023). 10.4244/EIJ-D-22-00537 PMC9853031 36073027

[170] G. Bonne, A. Muchir, G. Bonne, A. Muchir, “Cardiomyopathy caused by mutations in nuclear a-type lamin gene,” in *Cardiomyopathies - Types and Treatments* (IntechOpen, 2017); www.intechopen.com/chapters/52443.

[171] Y. A. Miroshnikova, T. Hammesfahr, S. A. Wickström, Cell biology and mechanopathology of laminopathic cardiomyopathies. J. Cell Biol. 218, 393–394 (2019). 30630866 10.1083/jcb.201805079 PMC6363443

[172] W. J. Tyler, The mechanobiology of brain function. Nat. Rev. Neurosci. 13, 867–878 (2012). 23165263 10.1038/nrn3383

[173] R. A. Stern, D. O. Riley, D. H. Daneshvar, C. J. Nowinski, R. C. Cantu, A. C. McKee, Long-term consequences of repetitive brain trauma: Chronic traumatic encephalopathy. PM R 3, S460–S467 (2011). 22035690 10.1016/j.pmrj.2011.08.008

[174] R. Gupta, N. Sen, Traumatic brain injury: A risk factor for neurodegenerative diseases. Rev. Neurosci. 27, 93–100 (2016). 26352199 10.1515/revneuro-2015-0017

[175] T. K. Mc Intosh, M. Juhler, T. Wieloch, Novel pharmacologic strategies in the treatment of experimental traumatic brain injury: 1998. J. Neurotrauma 15, 731–769 (1998). 9814632 10.1089/neu.1998.15.731

[176] M. Hemphill, S. Dauth, C. Yu, B. Dabiri, K. Parker, Traumatic brain injury and the neuronal microenvironment: A potential role for neuropathological mechanotransduction. Neuron 85, 1177–1192 (2015). 25789754 10.1016/j.neuron.2015.02.041

[177] K. C. Kasuba, A. P. Buccino, J. Bartram, B. M. Gaub, F. J. Fauser, S. Ronchi, S. S. Kumar, S. Geissler, M. M. Nava, A. Hierlemann, D. J. Müller, Mechanical stimulation and electrophysiological monitoring at subcellular resolution reveals differential mechanosensation of neurons within networks. Nat. Nanotechnol. 19, 825–833 (2024). 38378885 10.1038/s41565-024-01609-1 PMC11186759

[178] T. Grevesse, B. E. Dabiri, K. K. Parker, S. Gabriele, Opposite rheological properties of neuronal microcompartments predict axonal vulnerability in brain injury. Sci. Rep. 5, 9475 (2015). 25820512 10.1038/srep09475 PMC4377573

[179] D. E. Koser, A. J. Thompson, S. K. Foster, A. Dwivedy, E. K. Pillai, G. K. Sheridan, H. Svoboda, M. Viana, L. da F Costa, J. Guck, C. E. Holt, K. Franze, Mechanosensing is critical for axon growth in the developing brain. Nat. Neurosci. 19, 1592–1598 (2016). 27643431 10.1038/nn.4394 PMC5531257

[180] B. M. Gaub, K. C. Kasuba, E. Mace, T. Strittmatter, P. R. Laskowski, S. A. Geissler, A. Hierlemann, M. Fussenegger, B. Roska, D. J. Müller, Neurons differentiate magnitude and location of mechanical stimuli. Proc. Natl. Acad. Sci. U.S.A. 117, 848–856 (2020). 31882453 10.1073/pnas.1909933117 PMC6969492

[181] A. Carnicer-Lombarte, D. G. Barone, F. Wronowski, G. G. Malliaras, J. W. Fawcett, K. Franze, Regenerative capacity of neural tissue scales with changes in tissue mechanics post injury. Biomaterials 303, 122393 (2023). 37977006 10.1016/j.biomaterials.2023.122393

[182] E. Moeendarbary, I. P. Weber, G. K. Sheridan, D. E. Koser, S. Soleman, B. Haenzi, E. J. Bradbury, J. Fawcett, K. Franze, The soft mechanical signature of glial scars in the central nervous system. Nat. Commun. 8, 14787 (2017). 28317912 10.1038/ncomms14787 PMC5364386

[183] J. Lantoine, A. Procès, A. Villers, S. Halliez, L. Buée, L. Ris, S. Gabriele, Inflammatory molecules released by mechanically injured astrocytes trigger presynaptic loss in cortical neuronal networks. ACS Chem. Nerosci. 12, 3885–3897 (2021). 10.1021/acschemneuro.1c00488 34614352

[184] M. Shaughness, K. Byrnes, Assessment of the effects of stretch-injury on primary rat microglia. Mol. Neurobiol. 58, 3545–3560 (2021). 33763772 10.1007/s12035-021-02362-5

[185] M. C. Shaughness, N. Pierron, A. N. Smith, K. R. Byrnes, The integrin pathway partially mediates stretch-induced deficits in primary rat microglia. Mol. Neurobiol. 60, 3396–3412 (2023). 36856961 10.1007/s12035-023-03291-1

[186] J. Lantoine, T. Grevesse, A. Villers, G. Delhaye, C. Mestdagh, M. Versaevel, D. Mohammed, C. Bruyère, L. Alaimo, S. P. Lacour, L. Ris, S. Gabriele, Matrix stiffness modulates formation and activity of neuronal networks of controlled architectures. Biomaterials 89, 14–24 (2016). 26946402 10.1016/j.biomaterials.2016.02.041

[187] K. Krukowski, A. Nolan, M. Becker, K. Picard, N. Vernoux, E. S. Frias, X. Feng, M.-E. Tremblay, S. Rosi, Novel microglia-mediated mechanisms underlying synaptic loss and cognitive impairment after traumatic brain injury. Brain Behav. Immun. 98, 122–135 (2021). 34403733 10.1016/j.bbi.2021.08.210 PMC9119574

[188] N. Koushki, A. Ghagre, L. K. Srivastava, C. Molter, A. J. Ehrlicher, Nuclear compression regulates YAP spatiotemporal fluctuations in living cells. Proc. Natl. Acad. Sci. U.S.A. 120, e2301285120 (2023). 37399392 10.1073/pnas.2301285120 PMC10334804

[189] I. Baroja, N. C. Kyriakidis, G. Halder, I. M. Moya, Expected and unexpected effects after systemic inhibition of Hippo transcriptional output in cancer. Nat. Commun. 15, 2700 (2024). 38538573 10.1038/s41467-024-46531-1 PMC10973481

[190] G. Garoffolo, M. Casaburo, F. Amadeo, M. Salvi, G. Bernava, L. Piacentini, I. Chimenti, G. Zaccagnini, G. Milcovich, E. Zuccolo, M. Agrifoglio, S. Ragazzini, O. Baasansuren, C. Cozzolino, M. Chiesa, S. Ferrari, D. Carbonaro, R. Santoro, M. Manzoni, L. Casalis, A. Raucci, F. Molinari, L. Menicanti, F. Pagano, T. Ohashi, F. Martelli, D. Massai, G. I. Colombo, E. Messina, U. Morbiducci, M. Pesce, Reduction of cardiac fibrosis by interference with YAP-dependent transactivation. Circ. Res. 131, 239–257 (2022). 35770662 10.1161/CIRCRESAHA.121.319373

[191] K. P. Meng, F. S. Majedi, T. J. Thauland, M. J. Butte, Mechanosensing through YAP controls T cell activation and metabolism. J. Exp. Med. 217, e20200053 (2020). 32484502 10.1084/jem.20200053 PMC7398163

[192] Z. Wu, J. Su, F. Li, T. Chen, J. Mayner, A. Engler, S. Ma, Q. Li, K.-L. Guan, YAP silencing by RB1 mutation is essential for small-cell lung cancer metastasis. Nat. Commun. 14, 5916 (2023). 37739954 10.1038/s41467-023-41585-z PMC10516997

[193] A. A. Khalil, D. Smits, P. D. Haughton, T. Koorman, K. A. Jansen, M. P. Verhagen, M. Van Der Net, K. Van Zwieten, L. Enserink, L. Jansen, A. G. El-Gammal, D. Visser, M. Pasolli, M. Tak, D. Westland, P. J. Van Diest, C. B. Moelans, M. G. Roukens, S. Tavares, A.-M. Fortier, M. Park, R. Fodde, M. Gloerich, F. J. T. Zwartkruis, P. W. Zwartkruis, J. de Derksen, A YAP-centered mechanotransduction loop drives collective breast cancer cell invasion. Nat. Commun. 15, 4866 (2024). 38849373 10.1038/s41467-024-49230-z PMC11161601

[194] R. Islam, Z. Hong, YAP/TAZ as mechanobiological signaling pathway in cardiovascular physiological regulation and pathogenesis. Mechanobiol. Med. 2, 100085 (2024). 39281415 10.1016/j.mbm.2024.100085 PMC11391866

[195] A. Kaneda, T. Seike, T. Danjo, T. Nakajima, N. Otsubo, D. Yamaguchi, Y. Tsuji, K. Hamaguchi, M. Yasunaga, Y. Nishiya, M. Suzuki, J.-I. Saito, R. Yatsunami, S. Nakamura, Y. Sekido, K. Mori, The novel potent TEAD inhibitor, K-975, inhibits YAP1/TAZ-TEAD protein-protein interactions and exerts an anti-tumor effect on malignant pleural mesothelioma. Am. J. Cancer Res. 10, 4399–4415 (2020). 33415007 PMC7783735

[196] J. Barbazan, C. Pérez-González, M. Gómez-González, M. Dedenon, S. Richon, E. Latorre, M. Serra, P. Mariani, S. Descroix, P. Sens, X. Trepat, D. M. Vignjevic, Cancer-associated fibroblasts actively compress cancer cells and modulate mechanotransduction. Nat. Commun. 14, 6966 (2023). 37907483 10.1038/s41467-023-42382-4 PMC10618488

[197] V. S. Meli, H. Atcha, P. K. Veerasubramanian, R. R. Nagalla, T. U. Luu, E. Y. Chen, C. F. Guerrero-Juarez, K. Yamaga, W. Pandori, J. Y. Hsieh, T. L. Downing, D. A. Fruman, M. B. Lodoen, M. V. Plikus, W. Wang, W. F. Liu, YAP-mediated mechanotransduction tunes the macrophage inflammatory response. Sci. Adv. 6, eabb8471 (2020). 33277245 10.1126/sciadv.abb8471 PMC7717914

[198] Y. Shou, Z. Le, H. S. Cheng, Q. Liu, Y. Z. Ng, D. L. Becker, X. Li, L. Liu, C. Xue, N. J. Y. Yeo, R. Tan, J. Low, A. R. K. Kumar, K. Z. Wu, H. Li, C. Cheung, C. T. Lim, N. S. Tan, Y. Chen, Z. Liu, A. Tay, Mechano-activated cell therapy for accelerated diabetic wound healing. Adv. Mater. 35, e2304638 (2023). 37681325 10.1002/adma.202304638

[199] L. Gerosa, C. Chidley, F. Fröhlich, G. Sanchez, S. K. Lim, J. Muhlich, J.-Y. Chen, S. Vallabhaneni, G. J. Baker, D. Schapiro, M. I. Atanasova, L. A. Chylek, T. Shi, L. Yi, C. D. Nicora, A. Claas, T. S. C. Ng, R. H. Kohler, D. A. Lauffenburger, R. Weissleder, M. A. Miller, W.-J. Qian, H. S. Wiley, P. K. Sorger, Receptor-driven ERK pulses reconfigure MAPK signaling and enable persistence of drug-adapted BRAF-mutant melanoma cells. Cell Syst. 11, 478–494.e9 (2020). 33113355 10.1016/j.cels.2020.10.002 PMC8009031

[200] T. J. Aikin, A. F. Peterson, M. J. Pokrass, H. R. Clark, S. Regot, MAPK activity dynamics regulate non-cell autonomous effects of oncogene expression. eLife 9, e60541 (2020). 32940599 10.7554/eLife.60541 PMC7498266

[201] H. Zhan, S. Bhattacharya, H. Cai, P. A. Iglesias, C.-H. Huang, P. N. Devreotes, An excitable Ras/PI3K/ERK signaling network controls migration and oncogenic transformation in epithelial cells. Dev. Cell 54, 608–623.e5 (2020). 32877650 10.1016/j.devcel.2020.08.001 PMC7505206

[202] P. A. Gagliardi, B. Grädel, M.-A. Jacques, L. Hinderling, P. Ender, A. R. Cohen, G. Kastberger, O. Pertz, M. Dobrzyński, Automatic detection of spatio-temporal signaling patterns in cell collectives. J. Cell Biol. 222, e202207048 (2023). 37516918 10.1083/jcb.202207048 PMC10374943

[203] J. Ma, S. P. Iddir, S. Ganesh, D. Yi, M. J. Heiferman, Automated segmentation for early detection of uveal melanoma. Can. J. Ophthalmol. 59, e784–e791 (2024). 38768649 10.1016/j.jcjo.2024.04.003 PMC12388074

[204] R. J. Sullivan, J. R. Infante, F. Janku, D. J. L. Wong, J. A. Sosman, V. Keedy, M. R. Patel, G. I. Shapiro, J. W. Mier, A. W. Tolcher, A. Wang-Gillam, M. Sznol, K. Flaherty, E. Buchbinder, R. D. Carvajal, A. M. Varghese, M. E. Lacouture, A. Ribas, S. P. Patel, G. A. DeCrescenzo, C. M. Emery, A. L. Groover, S. Saha, M. Varterasian, D. J. Welsch, D. M. Hyman, B. T. Li, First-in-class ERK1/2 inhibitor ulixertinib (BVD-523) in patients with MAPK mutant advanced solid tumors: Results of a phase I dose-escalation and expansion study. Cancer Discov. 8, 184–195 (2018). 29247021 10.1158/2159-8290.CD-17-1119

[205] Z. Wolfe, J. C. Friedland, S. Ginn, A. Blackham, L. Demberger, M. Horton, A. McIntosh, H. Sheikh, J. Box, D. Knoerzer, B. Federowicz, T. J. Stuhlmiller, M. Shapiro, S. Nair, Case report: Response to the ERK1/2 inhibitor ulixertinib in BRAF D594G cutaneous melanoma. Melanoma Res. 32, 295–298 (2022). 35551160 10.1097/CMR.0000000000000830 PMC9245552

[206] E. I. Buchbinder, J. V. Cohen, G. Tarantino, C. G. Lian, D. Liu, R. Haq, F. S. Hodi, D. P. Lawrence, A. Giobbie-Hurder, D. Knoerzer, R. J. Sullivan, A phase II study of ERK inhibition by ulixertinib (BVD-523) in metastatic uveal melanoma. Cancer Res. Commun. 4, 1321–1327 (2024). 38683104 10.1158/2767-9764.CRC-24-0036 PMC11107576

[207] T. Grevesse, M. Versaevel, S. Gabriele, Preparation of hydroxy-PAAm hydrogels for decoupling the effects of mechanotransduction cues. J. Vis. Exp., 51010 (2014). 25225964 10.3791/51010 PMC4828013

[208] K. Adu-Berchie, Y. Liu, D. K. Y. Zhang, B. R. Freedman, J. M. Brockman, K. H. Vining, B. A. Nerger, A. Garmilla, D. J. Mooney, Generation of functionally distinct T-cell populations by altering the viscoelasticity of their extracellular matrix. Biomed. Eng. 7, 1374–1391 (2023). 10.1038/s41551-023-01052-y PMC10749992 37365267

[209] Z. Liu, Y.-R. Li, Y. Yang, Y. Zhu, W. Yuan, T. Hoffman, Y. Wu, E. Zhu, J. Zarubova, J. Shen, H. Nan, K.-W. Yeh, M. M. Hasani-Sadrabadi, Y. Zhu, Y. Fang, X. Ge, Z. Li, J. Soto, T. Hsiai, L. Yang, S. Li, Viscoelastic synthetic antigen-presenting cells for augmenting the potency of cancer therapies. Nat. Biomed. Eng. 8, 1615–1633 (2024). 39455719 10.1038/s41551-024-01272-w PMC12833746

[210] T. E. Brown, K. S. Anseth, Spatiotemporal hydrogel biomaterials for regenerative medicine. Chem. Soc. Rev. 46, 6532–6552 (2017). 28820527 10.1039/c7cs00445a PMC5662487

[211] J. Ahn, J. Ryu, G. Song, M. Whang, J. Kim, Network structure and enzymatic degradation of chitosan hydrogels determined by crosslinking methods. Carbohydr. Polym. 217, 160–167 (2019). 31079673 10.1016/j.carbpol.2019.04.055

[212] B. N. Narasimhan, M. S. Horrocks, J. Malmström, Hydrogels with tunable physical cues and their emerging roles in studies of cellular mechanotransduction. Adv. Nanobiomed. Res. 1, 2100059 (2021).

[213] L. Xiang, W. Cui, Biomedical application of photo-crosslinked gelatin hydrogels. J. Leather Sci. Eng. 3, 3 (2021).

[214] I. M. Tayler, R. S. Stowers, Engineering hydrogels for personalized disease modeling and regenerative medicine. Acta Biomater. 132, 4–22 (2021). 33882354 10.1016/j.actbio.2021.04.020

[215] Z. Li, H. Wang, K. Zhang, B. Yang, X. Xie, Z. Yang, L. Kong, P. Shi, Y. Zhang, Y.-P. Ho, Z.-Y. Zhang, G. Li, L. Bian, Bisphosphonate-based hydrogel mediates biomimetic negative feedback regulation of osteoclastic activity to promote bone regeneration. Bioact. Mater. 13, 9–22 (2021). 35224288 10.1016/j.bioactmat.2021.11.004 PMC8844702

[216] A. Räkel, A. Boucher, L.-G. Ste-Marie, Role of zoledronic acid in the prevention and treatment of osteoporosis. Clin. Interv. Aging 6, 89–99 (2011). 21594000 10.2147/CIA.S7282 PMC3095556

[217] H. Fleisch, Bisphosphonates: Mechanisms of action. Endocr. Rev. 19, 80–100 (1998). 9494781 10.1210/edrv.19.1.0325

[218] A. Rodrigo-Navarro, S. Sankaran, M. J. Dalby, A. del Campo, M. Salmeron-Sanchez, Engineered living biomaterials. Nat. Rev. Mater. 6, 1175–1190 (2021).

[219] W. G. Marshall, C. Gonzalez-Garcia, S. Trujillo, A. Alba-Perez, P. Childs, D. W. Shields, A. Tomlinson, R. Pettitt, B. Filliquist, P.-Y. Chou, M. J. Dalby, S. A. Corr, M. Salmeron-Sanchez, Bioengineering an osteoinductive treatment for bone healing disorders: A small animal case series. VCOT Open 6, e41–e51 (2023).

[220] L. Oliver-Cervelló, H. Martin-Gómez, C. Gonzalez-Garcia, M. Salmeron-Sanchez, M.-P. Ginebra, C. Mas-Moruno, Protease-degradable hydrogels with multifunctional biomimetic peptides for bone tissue engineering. Front. Bioeng. Biotechnol. 11, 1192436 (2023). 37324414 10.3389/fbioe.2023.1192436 PMC10267393

[221] L. Tao, Y. Gao, Y. Li, L. Yang, J. Yao, H. Huang, J. Yu, B. Han, B. Wang, Z. Liu, The preventive effect of photocrosslinked Hep/GelMA hydrogel loaded with PRF on MRONJ. BMC Oral Health 24, 1010 (2024). 39210345 10.1186/s12903-024-04792-8 PMC11363451

[222] J. Gačanin, C. V. Synatschke, T. Weil, Biomedical applications of DNA-based hydrogels. Adv. Funct. Mater. 30, 1906253 (2020).

[223] C. Wang, J. Zhang, Recent advances in stimuli-responsive DNA-based hydrogels. ACS Appl. Bio Mater. 5, 1934–1953 (2022). 10.1021/acsabm.1c01197 35138079

[224] M. M. F. A. Baig, W. L. Dissanayaka, C. Zhang, 2D DNA nanoporous scaffold promotes osteogenic differentiation of pre-osteoblasts. Int. J. Biol. Macromol. 188, 657–669 (2021). 34371047 10.1016/j.ijbiomac.2021.07.198

[225] M. Zhou, N. Liu, Q. Zhang, T. Tian, Q. Ma, T. Zhang, X. Cai, Effect of tetrahedral DNA nanostructures on proliferation and osteogenic differentiation of human periodontal ligament stem cells. Cell Prolif. 52, e12566 (2019). 30883969 10.1111/cpr.12566 PMC6536416

[226] D. Athanasiadou, N. Meshry, N. G. Monteiro, A. C. Ervolino-Silva, R. L. Chan, C. A. McCulloch, R. Okamoto, K. M. M. Carneiro, DNA hydrogels for bone regeneration. Proc. Natl. Acad. Sci. U.S.A. 120, e2220565120 (2023). 37071684 10.1073/pnas.2220565120 PMC10151614

[227] X. Yan, B. Yang, Y. Chen, Y. Song, J. Ye, Y. Pan, B. Zhou, Y. Wang, F. Mao, Y. Dong, D. Liu, J. Yu, Anti-friction MSCs delivery system improves the therapy for severe osteoarthritis. Adv. Mater. 33, e2104758 (2021). 34657320 10.1002/adma.202104758

[228] T. Yuan, Y. Shao, X. Zhou, Q. Liu, Z. Zhu, B. Zhou, Y. Dong, N. Stephanopoulos, S. Gui, H. Yan, D. Liu, Highly permeable DNA supramolecular hydrogel promotes neurogenesis and functional recovery after completely transected spinal cord injury. Adv. Mater. 33, e2102428 (2021). 34296471 10.1002/adma.202102428

[229] A. J. Engler, S. Sen, H. L. Sweeney, D. E. Discher, Matrix elasticity directs stem cell lineage specification. Cell 126, 677–689 (2006). 16923388 10.1016/j.cell.2006.06.044

[230] L. R. Smith, S. Cho, D. E. Discher, Stem cell differentiation is regulated by extracellular matrix mechanics. Physiology 33, 16–25 (2018). 29212889 10.1152/physiol.00026.2017 PMC5866410

[231] Z. Wei, M. Lei, Y. Wang, Y. Xie, X. Xie, D. Lan, Y. Jia, J. Liu, Y. Ma, B. Cheng, S. Gerecht, F. Xu, Hydrogels with tunable mechanical plasticity regulate endothelial cell outgrowth in vasculogenesis and angiogenesis. Nat. Commun. 14, 8307 (2023). 38097553 10.1038/s41467-023-43768-0 PMC10721650

[232] J. Na, C. Tai, Z. Wang, Z. Yang, X. Chen, J. Zhang, L. Zheng, Y. Fan, Stiff extracellular matrix drives the differentiation of mesenchymal stem cells toward osteogenesis by the multiscale 3D genome reorganization. Biomaterials 312, 122715 (2025). 39094522 10.1016/j.biomaterials.2024.122715

[233] D. Liu, G. Lu, B. Shi, H. Ni, J. Wang, Y. Qiu, L. Yang, Z. Zhu, X. Yi, X. Du, B. Shi, ROS-scavenging hydrogels synergize with neural stem cells to enhance spinal cord injury repair via regulating microenvironment and facilitating nerve regeneration. Adv. Healthc. Mater. 12, e2300123 (2023). 36989238 10.1002/adhm.202300123

[234] C. Vallejo-Giraldo, K. Krukiewicz, M. J. P. Biggs, Understanding the mechanobiology of gliosis may be the key to unlocking sustained chronic performance of bioelectronic neural interfaces. Adv. NanoBiomed Res. 2, 2100098 (2022).

[235] R. Amini, A. Bhatnagar, R. Schlüßler, S. Möllmert, J. Guck, C. Norden, Amoeboid-like migration ensures correct horizontal cell layer formation in the developing vertebrate retina. eLife 11, e76408 (2022). 35639083 10.7554/eLife.76408 PMC9208757

[236] H. Abuwarda, M. M. Pathak, Mechanobiology of neural development. Curr. Opin. Cell Biol. 66, 104–111 (2020). 32687993 10.1016/j.ceb.2020.05.012 PMC7578076

[237] S. M. Thomasy, B. C. Leonard, M. A. Greiner, J. M. Skeie, V. K. Raghunathan, Squishy matters – Corneal mechanobiology in health and disease. Prog. Retin. Eye Res. 99, 101234 (2024). 38176611 10.1016/j.preteyeres.2023.101234 PMC11193890

[238] J. Kaivola, K. Punovuori, M. R. Chastney, Y. A. Miroshnikova, H. Abdo, F. Bertillot, F. Krautgasser, J. D. Franco, J. R. W. Conway, G. Follain, J. Hagström, A. Mäkitie, H. Irjala, S. Ventelä, H. Hamidi, G. Scita, R. Cerbino, S. A. Wickström, J. Ivaska, Restoring mechanophenotype reverts malignant properties of ECM-enriched vocal fold cancer. bioRxiv 609159 [Preprint] (2024). 10.1101/2024.08.22.609159.

[239] M. W. Massidda, A. Demkov, A. Sices, M. Lee, J. Lee, T. T. Paull, J. Kim, A. B. Baker, Mechanical rejuvenation of mesenchymal stem cells from aged patients. bioRxiv 597781 [Preprint] (2024). 10.1101/2024.06.06.597781.

[240] S. K. Kureel, B. Blair, M. P. Sheetz, Recent advancement in elimination strategies and potential rejuvenation targets of senescence. Adv. Biol. 8, e2300461 (2024). 10.1002/adbi.202300461 37857532

[241] E. di Pasquo, A. J. O. Kiener, A. DallAsta, A. Commare, L. Angeli, T. Frusca, T. Ghi, Evaluation of the uterine scar stiffness in women with previous Cesarean section by ultrasound elastography: A cohort study. Clin. Imaging 64, 53–56 (2020). 32325262 10.1016/j.clinimag.2020.03.006

[242] M. T. Kuhlmann, S. Cuhlmann, I. Hoppe, R. Krams, P. C. Evans, G. J. Strijkers, K. Nicolay, S. Hermann, M. Schäfers, Implantation of a carotid cuff for triggering shear-stress induced atherosclerosis in mice. J. Vis. Exp., 3308 (2012). 22294044 10.3791/3308 PMC3462566

[243] J. Na, Z. Yang, Q. Shi, C. Li, Y. Liu, Y. Song, X. Li, L. Zheng, Y. Fan, Extracellular matrix stiffness as an energy metabolism regulator drives osteogenic differentiation in mesenchymal stem cells. Bioact. Mater. 35, 549–563 (2024). 38434800 10.1016/j.bioactmat.2024.02.003 PMC10909577

